# Variation in the Optical Properties of PEO-Based Composites via a Green Metal Complex: Macroscopic Measurements to Explain Microscopic Quantum Transport from the Valence Band to the Conduction Band

**DOI:** 10.3390/polym15030771

**Published:** 2023-02-02

**Authors:** Daron Q. Muheddin, Shujahadeen B. Aziz, Pshko A. Mohammed

**Affiliations:** 1 Medical Physics Department, College of Medicals & Applied Science, University of Charmo, Peshawa Street, Chamchamal, Sulaimanyah 46001, Iraq; 2 Hameed Majid Advanced Polymeric Materials Research Lab, Physics Department, College of Science, University of Sulaimani, Qlyasan Street, Kurdistan Regional Government, Sulaimani 46001, Iraq; 3 The Development Center for Research and Training (DCRT), University of Human Development, Sulaymaniyah 46001, Iraq; 4 Charmo Research Center, University of Charmo, Peshawa Street, Chamchamal, Sulaimanyah 46001, Iraq

**Keywords:** polymer composite, black tea PPHNL, green inorganic metal complex, XRD and FTIR, morphology, optical properties, quantum transport

## Abstract

In this study, a green chemistry method was used to synthesize polymer composites based on polyethylene oxide (PEO). The method of the remediation of metal complexes used in this study is an environmentally friendly procedure with a low cost. Zinc metal ion (Zn^2+^)-polyphenol (PPHNL) complexes were synthesized for two minutes via the combination of a black tea leaf (BTL) extract solution with dissolved Zn-acetate. Then, UV–Vis and FTIR were carried out for the Zn-PPHNL complexes in a liquid and solid. The FTIR spectra show that BTLs contain sufficient functional groups (O-H, C-H, C=O, C=C, C-O, C-N, and N-H), PPHNL, and conjugated double bonds to produce metal complexes by capturing the cations of Zn-acetate salt. Moreover, FTIR of the BTL and Zn–PPHNL complexes approves the formation of the Zn-PPHNL complex over the wide variation in the intensity of bands. The UV absorption spectra of BTL and Zn-PPHNL indicate complex formation among tea PPHNL and Zn cations, which enhances the absorption spectra of the Zn-PPHNL to 0.1 compared to the figure of 0.01 associated with the extracted tea solution. According to an XRD analysis, an amorphous Zn-PPHNL complex was created when Zn^2+^ ions and PPHNL interacted. Additionally, XRD shows that the structure of the PEO composite becomes a more amorphous structure as the concentration of Zn-PPHNL increases. Furthermore, morphological study via an optical microscope (OM) shows that by increasing the concentration of Zn-PPHNL in a PEO polymer composite the size of the spherulites ascribed to the crystalline phase dramatically decreases. The optical properties of PEO: Zn-PPHNL films, via UV–Vis spectroscopy, were rigorously studied. The *E_g_* is calculated by examining the dielectric loss, which is reduced from 5.5 eV to 0.6 eV by increasing the concentration of Zn-PPHNL in the PEO samples. In addition, Tauc’s form was used to specify the category of electronic transitions in the PEO: Zn-PPHNL films. The impact of crystalline structure and morphology on electronic transition types was discussed. Macroscopic measurable parameters, such as the refractive index and extinction coefficient, were used to determine optical dielectric loss. Fundamental optical dielectric functions were used to determine some key parameters. From the viewpoint of quantum transport, electron transitions were discussed. The merit of this work is that microscopic processes related to electron transition from the VB to the CB can be interpreted interms of measurable macroscopic quantities.

## 1. Introduction

The increasing demand for flexible polymer composites with narrow optical band gaps (OBGs) for application in solar cells and optoelectronic devices is in progress [1]. Manipulation of the OBGs of polymers toward minimization via the incorporation of inorganic complexes as additive fillers plays a crucial role. These metals possess high atomic masses and atomic density; as a result, they have widely been utilized in the solar energy industry [2,3]. These dopants, however, are not free of harmfulness as well as destructivity when introduced into the environment as waste materials. Despite their toxicity, metals have played a considerable role in the advancement of current science; for instance, in biology, chemistry, and engineering a high concentration level of metals results in environmental problems with serious implications for the environment and human health, which are extremely difficult to remedy [4,5].

To provide more biocompatible materials, a new method of green chemistry remediation has been followed to replace unsafe and hazardous heavy metal ions by a black tea extract solution. It is totally environmentally friendly and reduces health hazards for researchers as well as the whole environment. According to several reports, drinking tea contains antioxidant, anti-inflammatory, cancer-preventing, and heart-disease-reducing properties. The polyphenol (PPHNL)in black tea also contains amino acids, alkaloids, catechins (CTHs), theaflavins, their isomers, and other tea PPHNL derivatives. The components of black tea’s most precise chemical or molecular structures can be found elsewhere [4,5]. Along with a certain amount of CTH, the mixture also contains theaavins, thearubigins, and other CTH polymeric pigments that are important in producing metal complexes by capturing the cations of transition metals. A recent review by Drynan et al. found that black tea in an aqueous mixture is especially rich in PPHNL, PPHNL conjugates, and polymerized phenolic structures, which are the main components of tea. Additionally, black, green, and white teas include a variety of conjugated flavonoids [6]. Tea, which is produced from the leaves of the *Camellia sinensis* plant, is the most popular drink in the world. Depending on the degree of oxidation, tea can be divided into three main categories: unfermented green, moderately fermented Oolong, and fermented black tea [7]. The major components of tea extracts, as proven by HPLC observations in previous research, were PPHNLs, and their molecular representations can be seen elsewhere [8,9]. Earlier studies have also shown that green and black tea extract solutions included substantive amounts of conjugated double bonds, hydroxyl (OH), carboxylic groups, PPHNLs, and PPHNL conjugates [10,11]. These entities in tea dye contain a large number of conjugated ligands and functional groups that are crucial for the creation of metal complexes.

During the mass convey procedure recognized as sorption, a material is converted from a liquid into a solid, with the substance being attached by physical and/or chemical interactions. Sorption provides a less expensive choice compared to predictable methods owing to the large surface area, high sorption ability, and reactive surface of sorbents [12]. Brza, M.A. et al. investigated the capability of green chemistry to capture positively charged ions of transition metal salts as a replacement bioremediation for the production of organometallic-based products (e.g., copper chloride) [13]. In the same domain, organic–inorganic hybrid materials have undergone intensive studies and substantial progress has been achieved, exhibiting the fact that the field is an unquestionably major area of study; the results have shown clear evidence of the combination of organic and inorganic localized structures, which offer a great opportunity for preparing a variety of polymer composites. However, experts in this subject have suggested a variety of architectures, with various degrees of difficulty in practical execution, for handling the complexity associated with the drawing of materials with limited compatibility with one another [14].

Understanding and advancing future technologies depend greatly on fundamental research. Structure and composition manipulation bring up a wide range of possibilities for the development of innovative, high-performance materials. By the middle of this century, it is expected that global energy consumption will have tripled from its current level of 16 terawatts. Concerns over global warming and carbon emissions have motivated comprehensive research on solar cells as a sustainable replacement for fossil fuels. Consequently, recent years have seen an increase in interest in the technological development of photovoltaics [15]. As an alternative to purely organic solar cells (OSCs), organic–inorganic hybrid solar cells (HSCs), which combine organic and inorganic semiconductors, have received a considerable amount of research. Combining various inorganic semiconductors with organic semiconductors, as the active layer of devices, has been used to demonstrate this sort of solar cell [15,16]. Direct optical bad gap (OBG) semiconductor materials are widely used in solar cells and optoelectronic devices. Their significance is due to the fact that electrons can directly migrate to the conduction band after absorbing photon energy, whereas phonons are implicated in indirect OBG semiconductors, causing heating impacts and lowering device reliability. On the other hand, in broad OBG semiconductors interband transitions are more important processes compared to intraband transitions. Valence band to the conduction band, deep- and shallow-level transitions; both may occur in interband transitions [17]. When it comes to materials science research, molecular charge transfer (CT) systems have become an attractive and practical target, as they are tightly associated with magnetic change, transport photonic, dielectric, and structure properties. CT complexes play an important role in many electrophysical and optical phenomena, revealing important optical, electrical, and photoelectric features [18]. Knowing the optical absorption spectra in polymer composites is sufficient for finding out more about the OBG and band structure [19]. Furthermore, the optical characterization of polymer composites is helpful in identifying the role of defects and, consequently, in choosing polymeric materials for particular applications [20].

In our understanding, there is little published research on the optical characteristics of PEO-based composites. Polar polymers such as PVA, PEO, chitosan, MC, and PMMA are insulators in their pure state. In our previous work we showed that semiconductors and metallic powders modify the optical properties of insulating polymers [16,17,19,20]. Based on our recent study, metal complexes produced by green remediation are crucial for the band gap variation of polar polymers [13]. In this study, a green-synthesized metal complex will be added to PEO to reduce its OBG to a desired range that makes it suitable for optoelectronic applications.

The investigation of optical characteristics reveals that using green chemistry to fabricate polymer composites with excellent control over OBGs is a novel approach. Another merit of the current study is that microscopic processes related to electron transition from the VB to the CB in condensed matter physics can be interconnected with measurable macroscopic quantities. The findings imply that utilizing narrow-OBG PEO with superior film formation can eliminate limitations in longevity, cost, and flexibility, which limit the usage of conjugated polymers and help balance cost and performance.

## 2. Methodology

### 2.1. Materials and Sample Preparation

A facile and straightforward extraction methodology was followed to collect extracts (natural colorant tea) from black tea leaves by using distilled hot water [21]. Accordingly, 20 g of black tea leaves was added to 250 mL of distilled hot water, keeping the temperature at 90 °C, followed by cooling to ambient temperature. Subsequently, to remove undesired residue, Whatman filter paper (Whatman 41, cat. no. 1441) with a 20µm pore size was used.

**Figure 1:**
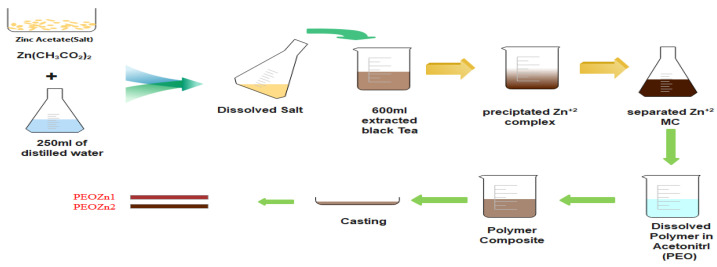
Schematic representing sample preparation.

Zinc acetate, Zn (CH_3_CO_2_)_2_.2H_2_O [MW = 219.51 g/mole], was purchased from Sigma-Aldrich. The Zn-PPHNL material was prepared upon the addition of 2.5 g of zinc acetate in to the pure colorant tea solution at 80 °C; subsequently, stirring of the mixture was carried out for 120 min. During the process of stirring, the dark tea solution changed to brown, accompanied by precipitate appearance in the form of a cloud at the bottom of the beaker as an indicator of Zn-PPHNL-based material formation. The Zn-PPHNL-based material preparation is illustrated in a scheme shown in Figure 1. The mixture was allowed to cool to room temperature and then the Zn-PPHNL-based material was separated using a centrifuge method. Finally, the Zn-PPHNL-based material was dispersed in 100 mL of distilled water after washing it several times. The method of the preparation of the Zn-PPHNL complex through a coordination approach, shown in Figure 1, follows a biosorption approach, which is an environmental remediation method; toxic materials can be captured and separated.

For the polymer composite and polymer films, PEO powder material with [MW = 2 × 10^6^ g/mole] was purchased from Sigma-Aldrich. The creation of solid polymer (SP) films based on PEO combined with Zn-PPHNL-based material was carried out by using the well-known solution cast process. Preparation of the PEO polymer solution begins by adding 80 mL of acetonitrile to three batches of 1 gm of PEO powder, followed by a magnetic stirrer stirring for 60 min. Then, 10 and 20 mL of the Zn-PPHNL-based material solution are separately added to the first and the second homogeneous PEO solutions in 10 mL steps, keeping the third batch a pure PEO solution. The mixtures were steadily stirred for 3 h until homogenous solutions were achieved. The samples were labeled as PEOZn0, PEOZn1, and PEOZn2 for pure samples and PEOs filled with 0, 10, and 20 mL of Zn-PPHNL-based material solution, respectively. The solid polymer composite films were made by adding the solutions into several dry Petri dishes, allowing them to dry for a week at room temperature. About 5 films from each sample were prepared to guarantee quantitative results’ reproducibility, as well as to allow us to choose the optimum films. Further drying of the films was carried out in a desiccator filled with a proper quantity of blue silica gel prior to characterizations.

### 2.2. Spectroscopic Studies

Within a wavenumber range of 400 to 4000 cm ^−1^, and a resolution of 2 cm^−1^, the samples were analyzed by using a Spotlight spectrophotometer (*Nicolet iS10 FTIR*). The UV–Vis Perkin Elmer double-beam UV–Vis–NIR spectrometer (Lambda 25) absorption was used to acquire the UV–Vis spectra of the synthesized series of samples, including PEOZn0, PEOZn1, and PEOZn2 films. X-ray diffraction (XRD) spectra were recorded in order to understand the structural profiles for all of the synthesized films. Under the condition of glancing angles in the range of (10° ≤ 2θ ≤ 80°) in a 0.1° step size, an X’Pert pro diffractometer (Pan Analytical) was used. Further characterization comprises the optical micrograph (OM) images captured of the surface microstructures of the films. The morphology of the composites was characterized by an optical microscope (*Am Scope, Fixed Microscope Adapter FMA 050*) with a digital camera (14 MP APTINA COLOR CMOS ULTRA-FINE COLORE ENGINE INSIDE).

## 3. Results and Discussion

### 3.1. XRD Analysis

**Figure 2:**
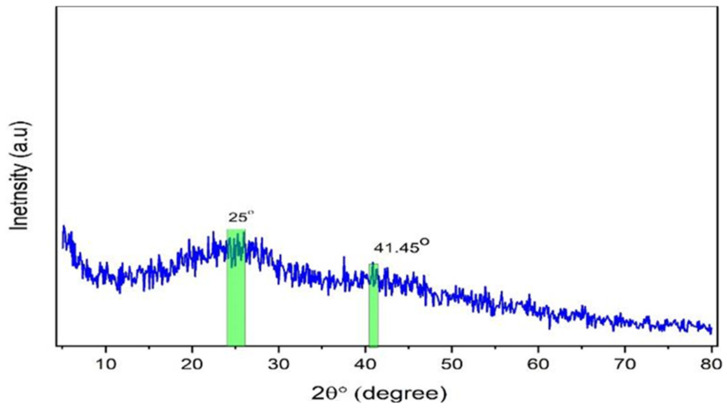
XRD pattern for the synthesized zinc metal complex.

**Figure 3:**
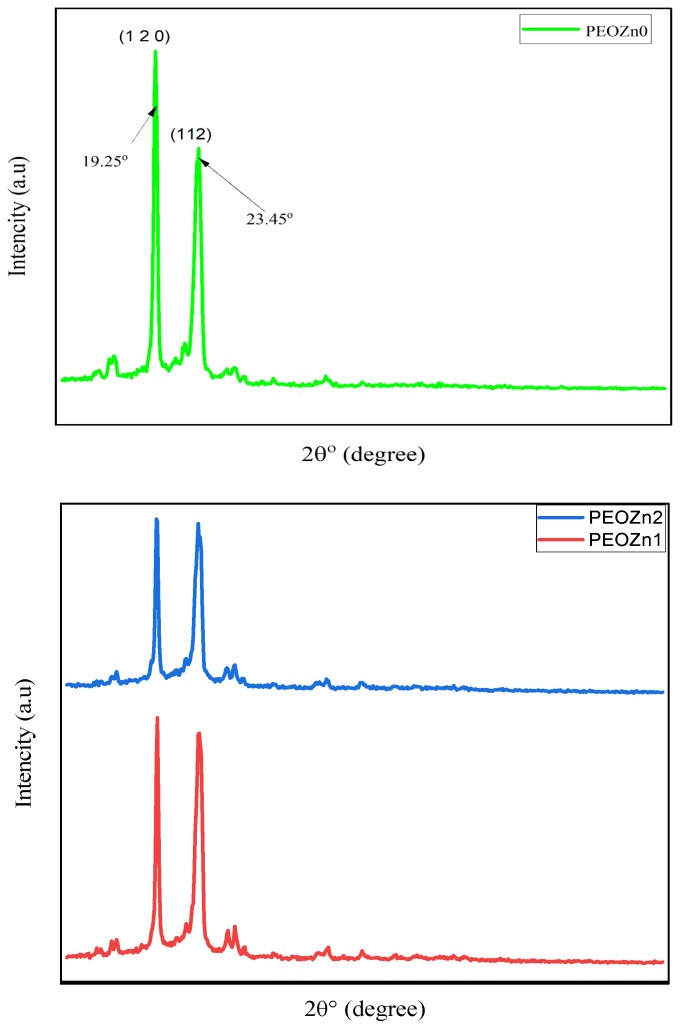
XRD pattern for pure PEOZn0, PEOZn1, and PEOZn2.

An X-ray diffractometer (XPERT-PRO) with a source of Cu kα and wavelength (0.154 nm) was employed to record the polymer films’ XRD patterns at room temperature, with Bragg’s angle (2θ) in the range of 10 to 80° and a scan rate of 2 min^−1^. The XRD pattern of the Zn-PPHNL complex is demonstrated in Figure 2. The structure of the generated Zn-PPHNL complex is predominantly amorphous because there are no crystalline peak appearances over the entire range of 2θ°. From 2θ = 25° to 2θ = 41.45°, only the hump is apparent (Figure 2). Figure 3 displays the X-ray spectra for PEO films. Pure PEO exhibits a sharp peak of intensity at 19.25°, another maximum at 23.45°, indicating high-intensity diffraction peaks, and some tiny bumps at higher angles. As a general principle, as a polymer crystallizes organized structures appear hierarchically at different length scales. The folding of a polymer chain typically leads to the formation of a folded-chain lamellar crystal with a distinct period over the molecular dimension. This chain-folding structure experiences free development in two lateral dimensions. There is, however, a restriction in the chain extension propagation, where the majority of the flaws are concentrated on the folding surfaces [22]. The peaks that appeared in Figure 3 were caused by strong intermolecular interactions between PEO chains connected by hydrogen bonds and the order of the polyether side chains [23,24,25]. This result is similar to earlier results from [26], which showed that the crystalline peaks for a pure PEO sample occurred at these specific angles. From the characteristic diffraction peaks, one can recognize whether the surfaces of the PEO polymers are crystalline or semicrystalline [27,28,29]. Looking at PEO as a linear and semicrystalline polymer, its structural components, including the C-H, C-C, and C-O bonds, lead to keeping the PEO polymer’s crystalline structure, chemically as well as electrochemically [30]. Rajeh et al. recently stated that peaks at about 22° and 18° correspond to the (112) and (120) planes [22]. It is clearly observed that as a convenient amount of Zn-PPHNL (20 mL) is added to the matrix of a PEO polymer, the relative intensities of the peaks of the XRD decline and are accompanied with the growth of the amorphous behavior of composite films. The declining of peak intensities is caused by the structural reformation ofO-polar groups in the chemical formation of PEO, creating desired interaction with the dopants [31]. In general, there are two kinds of polymers: crystalline and amorphous polymers. The former possess reflected planes, thereby giving miller indices because of a compact crystal assembly of stereo-regular chains. On the other hand, amorphous polymers experience a rubbery or glassy behavior [31]. Bandara et al. observed spherulites in PEO that blended with an Al_2_O_3_ filler as a result of the existence of a crystalline phase [32].

### 3.2. FTIR Analysis

**Figure 4:**
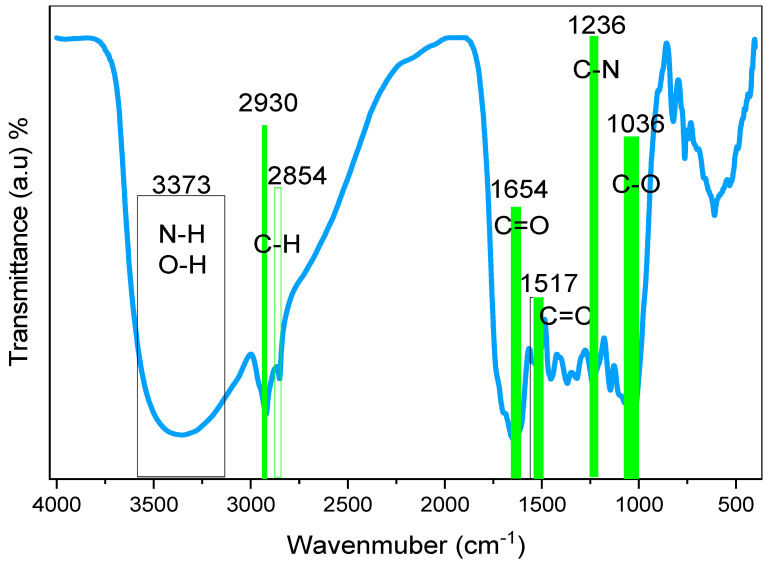
FTIR spectrum of black tea leaves.

FTIR was used to identify and describe the functional groups of BTL extract solution. The functional groups of the BTL sample could be obtained by analyzing the spectral range between 400 and 4000 cm^−1^, since it allows for the identification of the vibrational frequency of the chemical bonds that make up both inorganic and organic molecules. Absorption at functional groups occurs as a result of vibrations in a certain frequency band and exhibits a clear feature of IR absorption [33]. Figure 4 displays the FTIR spectra of BTL extracts. The broad band detected at 3373 cm^−1^ is linked to wide O-H and N-H stretching vibrations of PPHNL [34,35]. Previously, there was confirmation that the stretching modes of N-H in (secondary and primary) amide and amine, O-H in phenols, alcohols, and carboxylic acids, occupy the range of 3411 to 3370 cm^−1^. Moreover, the C-H stretching vibrations of aliphatic and carboxylic groups have a peak position between 2930 cm^−1^ and 2854 cm^−1^ [36]. Importantly, the carbonyl bonds (C=O) of catechins’ PPHNL and flavonoids have the mode of stretching vibrations, giving peaks at 1654 cm^−1^ [37,38]. Interestingly, PPHNL and caffeine have the strong appearance of a peak for the alkene group (C=C), stretching at 1517 cm^−1^, and C=C vibrations of aromatic compounds peak at 1517 cm^−1^. The stretching mode within the aromatic compounds is identified at 1480 cm^−1^ [39,40]. Within the 1440 to 1410 cm^−1^ range, a new wideband is recorded for both carbonate C-O stretch vibrations and carboxylic acid O-H in a bent plane [41]. For a number of compounds, including carboxylic acids, esters, and alcohols, a sharp and strong peak lies at 1036 cm^−1^; identifying the stretching modes of C-O and the stretching vibration peaks around1236 cm^−1^ originates from the C-N vibrations in aliphatic amine groups [39,40]. The recorded FTIR spectra in the present study for BTLs is relatively analogous to those reported in the literature for green and black teas [33,36,41,42,43,44,45]. Both the bending and stretching vibrations in pyrimidine, imidazole, carbonyl, and methyl groups are in good accordance with the bands in the caffeine spectra that change between 1700 cm^−1^ and 400 cm^−1^ [46,47,48].

**Figure 5:**
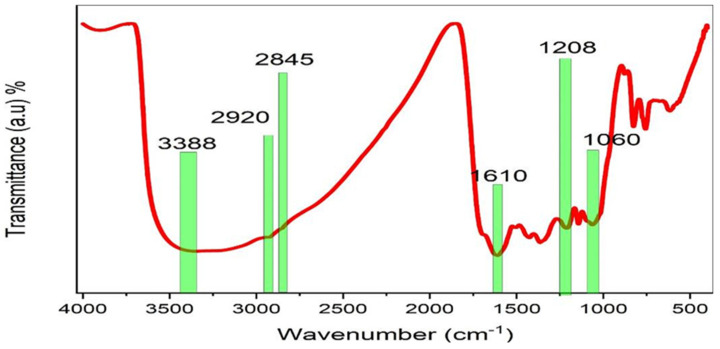
FTIR of the Zn-PPHNL metal complex.

According to published research, PPHNL and metal cations can combine to produce cation–PPHNL complexes [48,49,50]. Additionally, Al^+3^, Cd^+2^, and Ce^+2^-PPHNL complexes were reported in [50,51]; utilizing a BTL extract solution, the authors stated that when metal ions mix with PPHNL, a metal–PPHNL complex is created, which can be confirmed by the appearance of a colloidal suspension and green solution at the bottom and top of the container, respectively [10,11,52,53,54]. Figure 5 demonstrates the Zn-PPHNL combination’s FTIR spectrum. The peaks at 2920.24 and 2845.93 cm^−1^ of the Zn-PPHNL complex have almost completely vanished, as can be seen by the comparison of Figure 4 and Figure 5. This is in agreement with the theory that implies that Zn^2+^ ions’ attachment to PPHNL reduces their vibrations and increases their weight. Additionally, the peaks in Figure 5, particularly those in the spectral region between 1700 and 400 cm^−1^, were changed. Caffeine and PPHNL in tea extracts interact with metal ions as previously mentioned, and many complexes are involved in the chemistry of this interaction between Zn^2+^ ions and extract solutions of tea. Both the Zn-PPHNL and Zn^2+^-caffeine complexes are well-expected.

**Figure 6:**
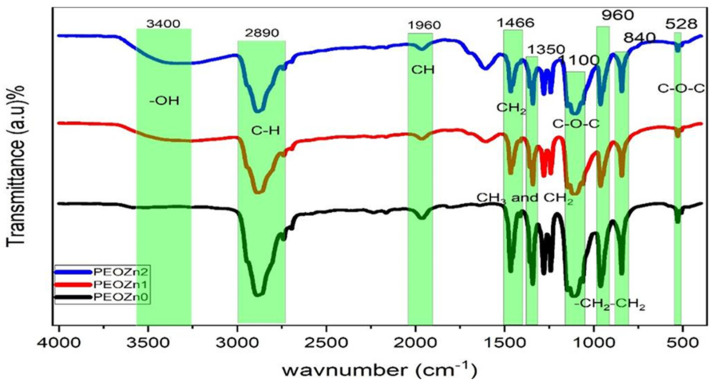
FTIR spectra of neat PEO and PEO composite samples.

**Figure 7:**
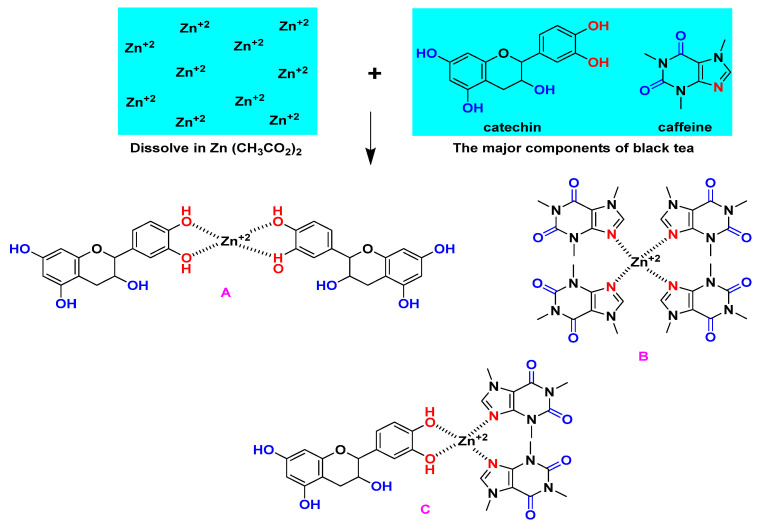
The creation of the Zn-PPHNL metal complex as proposed.

FTIR spectroscopy can identify complex interactions between PEO and dopants, as illustrated in Figure 6. The peaks in the FTIR spectrum of pure PEO are associated with a number of vibrational modes; for instance, the broad peak at about 3400 cm^−1^ was related with the OH stretching of Zn-PPHNL. The sharp peak at about 2890 cm^−1^ belongs to the C-H stretching mode [31,55,56,57]. The peak at around 1961 cm^−1^ corresponds with the CH asymmetric stretching of CH_3_ [58]; the 1466 cm^−1^ peak is due to the asymmetric bending of CH_2_. The 1359 to 1350 cm^−1^ peak is associated with CH_2_ wagging and CH_3_ bending. The peaks at about 1100, 960, and 840 cm^−1^ occur as a result of C-O-C stretching and -CH_2_-CH_2_ rocking, C-O-C vibration modes, and CH_2_ rocking, respectively [59,60]. Overall, the peaks become less intense in the PEOZn0 sample compared to PEOZn2, which is an indication of polymer complex formation [61]. The peaks’ positions and intensities start to shift when the concentration of Zn-PPHNL in the samples increases. As a result, Figure 7 suggests complex formations of Zn^+2^ with the PPHNL and caffeine of extract tea solutions according to a previous study and the FTIR analysis of the current work.

### 3.3. Absorption Study

**Figure 8:**
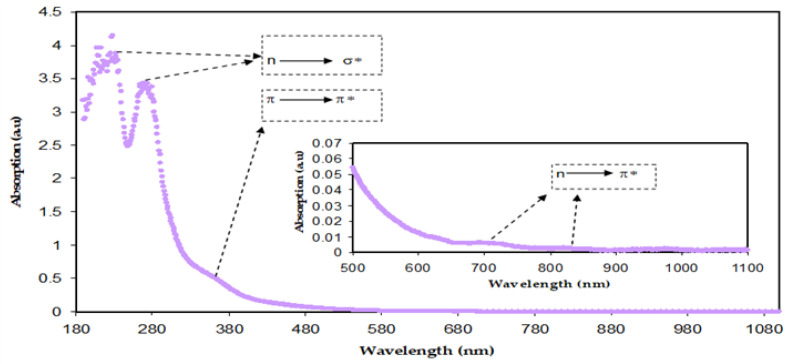
Absorption spectra of diluted BT solution.

Figure 8 demonstrates the absorption spectra of both BTL extract solution and Zn-PPHNL suspended particles. There are various types of electronic transition that can be seen. When the molecules absorb photons in the visible and ultraviolet regions their electrons excite from *σ*, *π*, and *n*-orbitals to higher energy states [21]. The electronic transitions associated with ultraviolet (180–260 nm) causes the electronic transition of n→σ*, whereas transitions such as *π*→π* and *n*→π* of CTH, methylxanthines, and caffeine demand considerably less energy; therefore, they occur at higher wavelengths [62,63,64]. Marzuki et al. observed the same absorption spectra for green tea that had been extracted using an ethyl acetate solvent [65]. According to research, conjugated systems with alternating double bonds are a crucial class of materials for optoelectronic applications because of their *π*-excessive properties [66]. The change to longer wavelengths implies that the samples of doped polymer have a smaller OBG. A semiconducting nature or *π*-conjugated polymers with a low band gap are widely used in industrial applications such as molecular electronics, nonlinear optical devices, organic light-emitting diodes, organic solar cells, memories, and energy storage [67]. The *π*-delocalization within the polymer chains in the films is due to the chemical structure of the components that are extracted from green tea. The tea samples are chemically complex due to the existence of many organic compounds, including PPHNLs, alkaloids, amino acids, glucides, proteins, volatile compounds, minerals, and trace elements [9]. PPHNLs are considered the most interesting group of compounds in both green and black tea leaves [68]. The primary components of PPHNLs are enriched by OH groups and conjugated double bonds [69]. The most frequently confirmed component structures of extract tea solutions can be observed elsewhere [8,9,68,69]. An earlier study stated and confirmed the absorbance band appearance within 200–350 nm, resulting from the electronic transition of *n*–π* in caffeine, methylxanthines, and CTH. At 277 nm, the caffeine possesses an absorption band [70,71].

**Figure 9:**
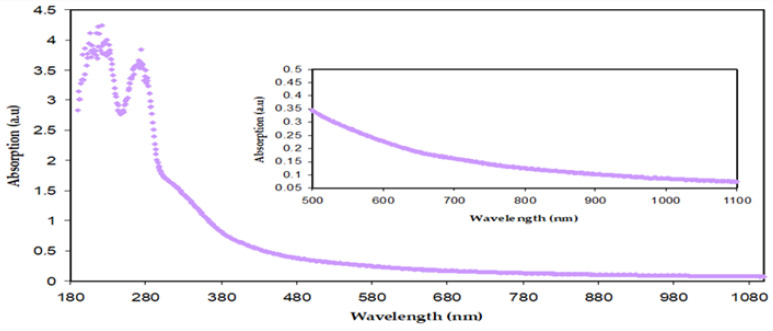
Absorption spectra for Zn-PPHNL.

Thus, the extract tea solution consists of sufficient hydroxyl (OH), carboxylic (C=O) groups, conjugated double bonds, PPHNLs, and PPHNL conjugates, which are useful for the creation of complexes with transition metal salts and polymer functional (polar) groups [10]. The absorption peaks seen at high wavelengths (380–700 nm) of Zn-PPHNL (see Figure 9) are associated with the presence of *π* electrons and the formation of charge transfer molecular systems [64]. Due to complex formation among tea PPHNLs and Zn cations, the absorption spectra of Zn-PPHNL is enhanced to (0.1) compared to that of the tea extract solution (0.01);however, the absorption spectrum is still weak when visible. The result is in good agreement with the previous reports that use metal–PPHNL complexes through green chemistry methods. Nevertheless, by adding this complex material to some polymers it can be used as a harvesting structure in optoelectronic applications because of its ability to absorb a wide band of solar radiation.

### 3.4. Morphological Study of PEO Composites

**Figure 10:**
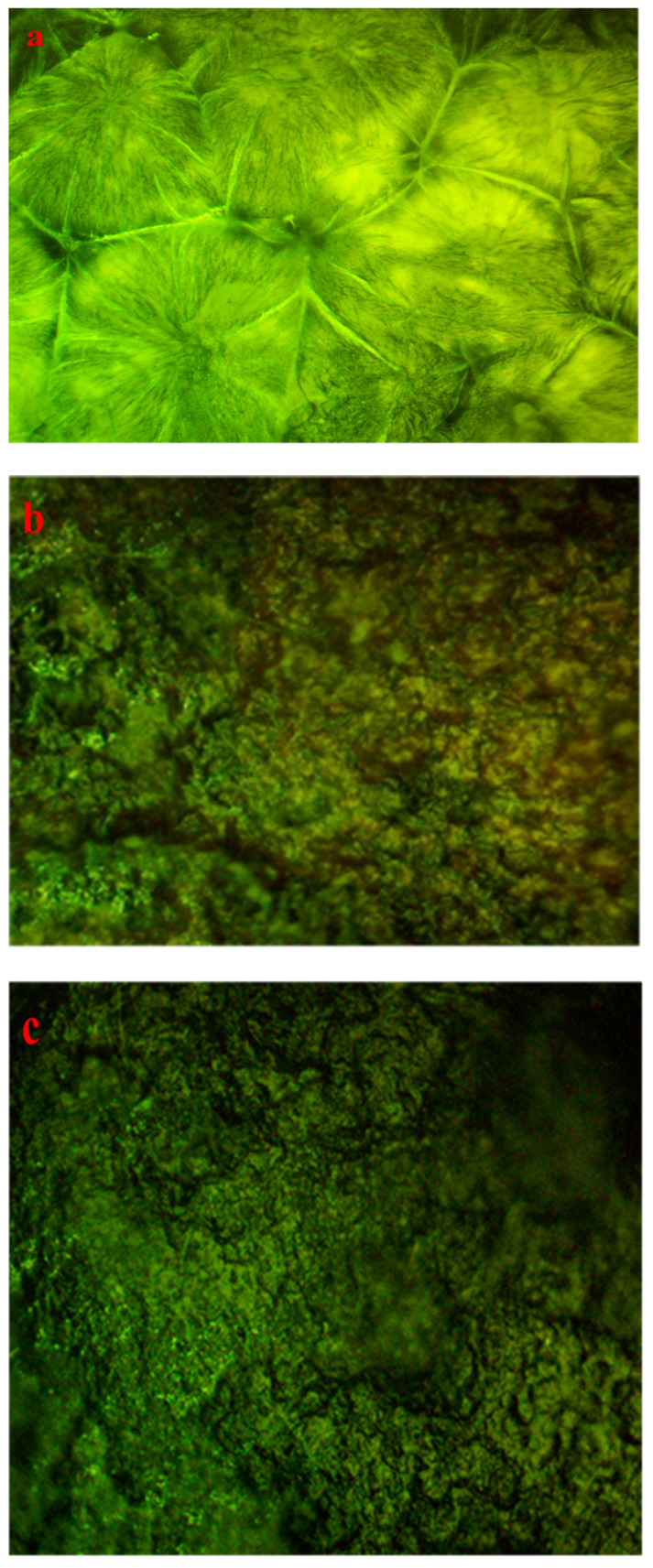
Optical microscope pictures for (**a**) PEOZn0, (**b**) PEOZn1, and (**c**) PEOZn2 films.

Some factors, including the production method, temperature, concentration, and dopant material, affect the structural morphology of PEO films. Optical micrographs, shown in Figure 10a–c, can be utilized to analyze morphological studies on PEO-based composites [72,73,74]. From Figure 10a, several large-diameter spherulites (SPHLs) can be seen as particular features of pure PEO films. The literature states that random nucleation creates spheroids, which continue through radial development until they intersect with one another at borders. Crystalline polymers with bendable chains exhibit this type of morphological behavior [75]. On the other hand, the accumulation of 10 mL of Zn-PPHNL to pure PEO resulted in the total fracturing of SPHL structures into small-sized SPHLs and dark spots that promote the amorphous structure [76], as shown in Figure 10b. One probable reasonfor this deformation of composite films is the random distribution of Zn-PPHNL in a PEO’s structure [32]. Increasing the amount of Zn-PPHNL to 20 mL leads to an increase in the number of SPHLs, and their sizes become smaller compared to a pure film, as shown in Figure 10a. Furthermore, the PEO chains prevent the crystalline lamellae from growing through a particular pattern, causinga decrease in the degree of crystallinity [73].

## 4. Absorption Coefficient Study

**Figure 11:**
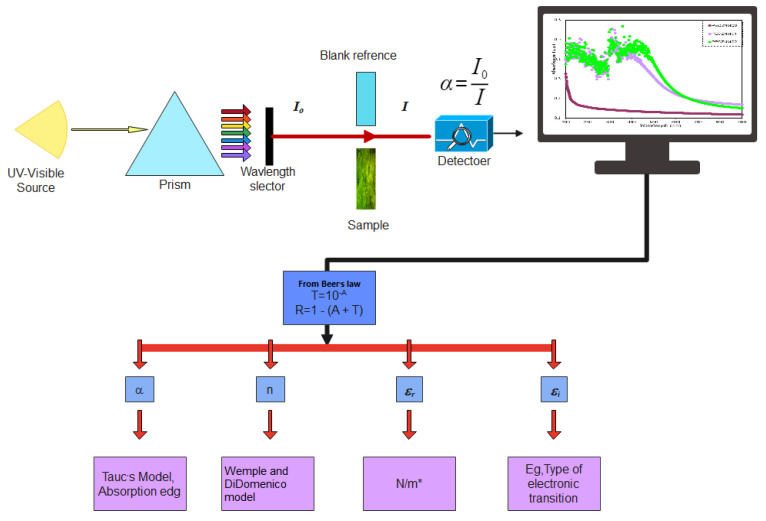
Flowchart of the sample’s optical properties.

In this work, the UV–Vis spectra were used to study several optical parameters, as shown in Figure 11.

**Figure 12:**
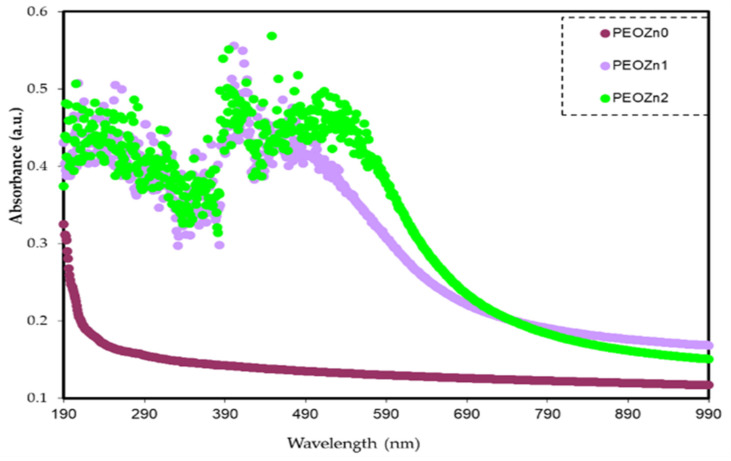
Absorption spectra for PEO composites.

The absorption spectra of pure PEO and PEO-based composites are demonstrated in Figure 12. It is obvious that pure PEO does not show any absorption peak in the visible spectrum. The polymer film remains transparent at this spectral range as the incident light is incapable of establishing electronic transition. In contrast, the absorption of PEO composites increases due to the additives, which aid in reducing the OBG of PEO. In polymer composites photon energy is substantially absorbed by the materials in the ultraviolet and visible ranges; the absorption leads to electron transitions in the *σ*, *π*, and *n*-orbitals to higher energy states [21]. Since the vast majority of the optical transitions are caused by impurities that have energies lain throughout the visible spectrum, the defects are regarded as color centers [77]. Because of the strong *π*→π* interactions, the absorption spectra of PEO:Zn-PPHNL solid films are more widened than those of Zn-PPHNL solutions. This kind of interaction is explained by the *π*→π* staking and packing effect under solid-state conditions [67]. In addition, the presence of *π*-delocalization along the polymer chain can be attributed to strong shifts towards longer wavelengths. This hypothesis is significantly confirmed by the absence of absorption peaks in a pure PEO film [78,79]. The absorption of PEO:Zn-PPHNL composite films begins in the near-infrared region and extends across the full UV–Vis region; the absorption maxima of a PEO:Zn-PPHNL film display a red shift, indicating the presence of interchain interactions in the solid structure [66]. Materials with amendable absorbance in the lower–mid visible spectrum are important for applications such as optoelectronics and optical sensors.

The outcomes of this study could be of great potential in encouraging research on light harvesting in the visible region [80]. Furthermore, the findings here suggest that metal complexes, as compared to multiwalled carbon nanotubes (MWNTs), are more effective in modifying the optical characteristics of polymers [81].

**Figure 13:**
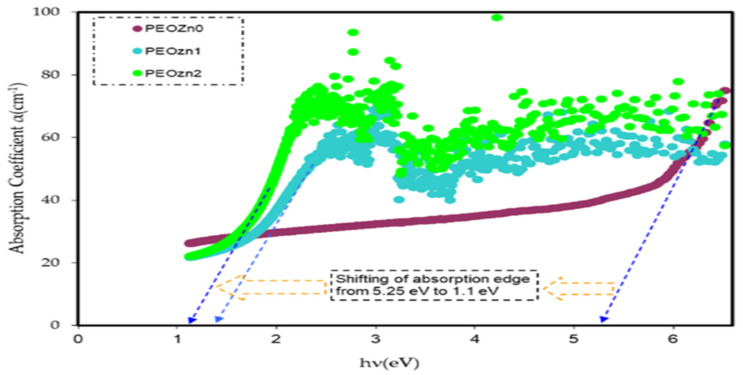
Absorption coefficient spectra against photon energy for PEO composites.

Interband absorption is a well-known technique for investigating the transition of electrons between the solid bands. Fundamental absorption, which appears as a rapid change in the material spectrum, is referred to as the absorption edge. This is in strong association with either band-to-band or exciting transitions and is regarded as a suitable indicator of OBG energy. The absorption coefficient is expressed by *α*(*υ*), which measures how quickly the intensity of the incident light decreases in accordance to a medium length [82,83]. The Beer–Lambertequation is applied to calculate the *α(υ)* from the absorbance spectra, *A*(*υ*), at the equivalent frequency (*v*):

(1) $$\alpha \left(v\right)=\frac{2.303}{d}log\left(\frac{I_{o}}{I}\right)=\frac{2.303}{d}A\left(v\right)\;$$

where $I_{o}$ is incident beam intensity, I is transmitted beam intensity, and *d* is sample thickness. The incident beam intensity *I_o_*, equals the sum of the intensities of the reflected, absorbed, and transmitted beams (labeled as *I_R_*, *I_A_*, and *I_T_*, respectively) when they hit the second medium surface, or as follows:

*I_o_* = *I_T_* + *I_A_* + *I_R_* (2)

the above equation can be written as follows:

*A* + *T* + *R* = 1 (3)

where *A*, *T*, and *R* represent, respectively, the absorptivity, *I_A_*/*I_o_*, transmissivity, *I_T_*/*I_o_*, and reflectivity, *I_R_*/*I_o_*, or the fractions of incident radiation which are transmitted, absorbed, and reflected by a material. The *T* value can be calculated using Beer’s equation (i.e., *T* = 10^−*A*^), where *A* is the raw absorption and reflectance, *R*, is required for the calculation refractive index, determined from Equation (3). The study of optical absorption, particularly the absorption edge, is a key tool for understanding materials’ electronic structures and the existence of direct and indirect transitions [13,84]. Table 1 includes the values of absorption edges as well as the absorption edge values of polymer films computed by extrapolating the linear part of the ordinate to zero. It is clear that adding Zn-PPHNL causes the absorption edge to reduce remarkably for pure PEO, from 5.25 eV to 1.1 eV for PEO:Zn-PPHNL samples, shifting toward low photon energy (see Figure 13). The substantial change in the absorption edge implies a significant change in theband structure polymer composites due to the creation of new localized states in the mobility gap [85]. In other words, absorption edge data indicate that a polymer with wide band gap is converted into a narrow OBG, which plays a significant role in the development of organic solar cells and optoelectronic devices [86,87].

### 4.1. Refractive Index Study

The refractive index is strongly linked to the polarizability of ions and the local field inside the substance. The parameter is crucial for purposes of integrated optical devices, such as modulators, filters, and switches [88]. Along with the optical dielectric constant, it is regarded as a significant characteristic in the design of new compounds for various optical electrical applications. When a beam of electromagnetic light transmits through a material, reflectance and absorption can be used to determine the refractive index of a material, which expresses as the following:

n*(*λ*) = *n*(*λ*) + *k*(*λ*) (4)

where n* is the complex refractive index, the real part of the refractive index is *n*, which is related to the actual velocity, and *k* is the extinction coefficient. Fresnel formulae are helpful in calculating the refractive indices of pure PEO films and PEO films doped with Zn-PPHNL by using the values of reflectance, *R*, and the optical extinction coefficient, *k* = *αλ*/4*πd*; *α* and *λ* sequentially stand for the absorption coefficient and the wavelength, and d is the sample thickness [89]:

(5) $$n\left(\lambda \right)=\sqrt{\frac{4R}{{\left(1-R\right)}^{2}}-k^{2}}+\left(\frac{1+R}{1-R}\right)\;$$

**Figure 14:**
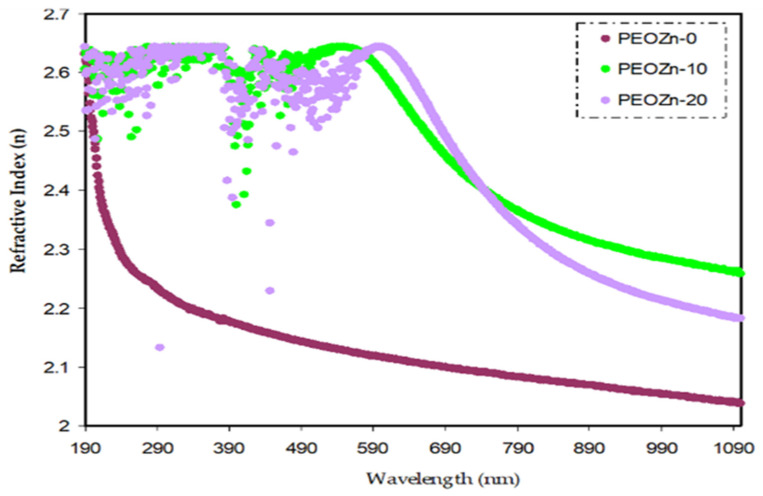
Pure and doped PEO films’ refractive index (*n*) vs. wavelength.

When a light beam is guided through an optical medium, the refractive index *n*(*λ*) defines how transparent materials are. For materials that are entirely transparent *n*(*λ*) approaches zero, and positive values indicate that light is being absorbed. The dispersion curve of the refractive index, *n*(*λ*), for pure PEO and PEO doped with Zn-PPHNL is shown in Figure 14. The doped samples exhibit larger *n* values and display notable dispersion. The results indicate that adding a Zn-PPHNL complex to a PEO polymer increases the refractive index from 2.05 to about 2.27. This is most likely due to the space charge creation in the Zn-PPHNL complex. As previously mentioned, altering the *n* value of materials in accordance with wavelength is important for changing their optical properties, with their dispersion being quite significant from an application viewpoint. The most significant optical materials have a refractive index, *n*, between two and three, as visible light absorbs in the upper atomic layers of the material [90,91]. The refractive index value (typically > 1.65) for PEO/Zn-PPHNL metal complex composite films makes them suitable basic structures for photovoltaic and optical devices in applications such as Bragg gratings, solar cells, waveguide-based optical circuits, and photonic crystals [92].

### 4.2. Wemple and DiDomenico (W–D) Model

Wemple and DiDomenico’s (W–D) single-oscillator model can be used to investigate refractive index dispersion in the normal region [93]. In order to conduct the exploration, a parameter of dispersion energy (*E_d_*) is introduced as a measure of the force of the optical interband transition. *E_d_* is strongly linked to chemical bonding and combines the charge distribution and coordination number in each unit cell [94]; however, the parameter of a single oscillator (*E_o_*) is proportional to the oscillator energy. Equation (6) represents a semi-empirical relationship that links the photon energy, *hυ*, and refractive index, *n*, below the interband absorption edge:

(6) $$n^{2\;}-1=\frac{E_{d}\;E_{o}}{\left[{E_{o}}^{2}-{\left(hv\right)}^{2}\right]}\;$$

**Figure 15:**
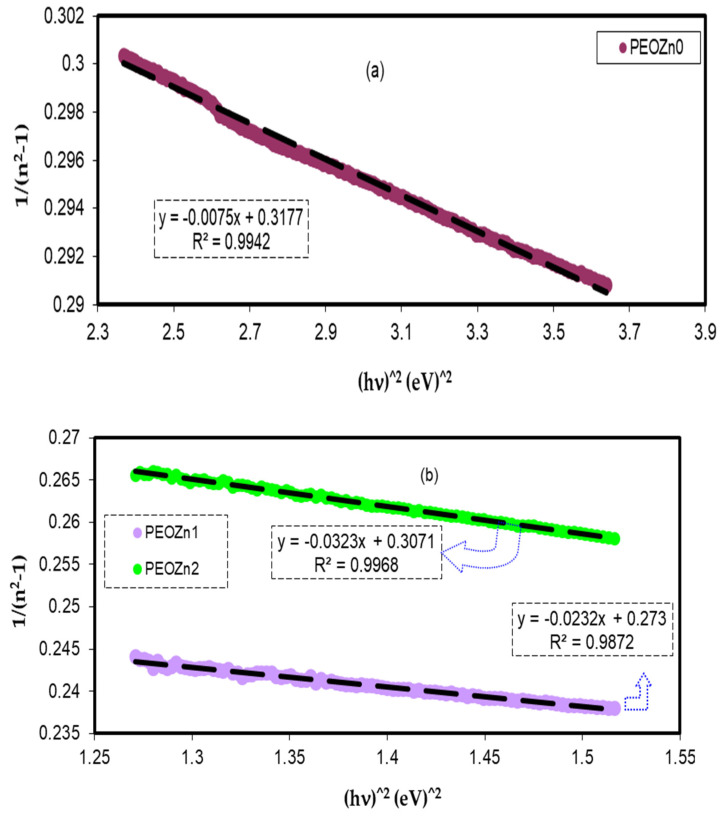
Variant 1/*n*^2^ − 1 against photon energy (*hv*)^2^ for (**a**) PEOZn0 and (**b**) PEOZn1, and PEOZn2 samples.

As shown in Figure 15a,b, the data on the plots of 1/*n*^2^ − 1 against (*hυ*)^2^ were matched with linear regression lines to the values of *E_d_* and *E_o_* from the intercept and slope, respectively. The determined values of *E_d_* and *E_o_* are shown in Table 2. *E_d_* and *E_o_* values decrease as the concentration of the Zn-PPHNL complex solution increases. There is a link between *E_o_* and the OBG, *E_g_*. The refractive index, *n*, values and the single effective oscillator energy, *E_0_*, are in disagreement with those obtained from Tauc’s law [95]; however, for the current films, empirically the values of *E_o_* are not matched with the direct *E_g_* (i.e., *E_o_* ≈ *E_g_*), as shown in Section 4.4.

### 4.3. Optical Dielectric Constant Study

Regarding polymer composites, the optical transition mainly corresponds to changes in the optical dielectric constant, which characterizes the possibility that an electron could lose energy while passing through the surface of the bulk material [96]. The dielectric constant of the materials (as expressed in Equation (7)) consists of a real part, *ԑ_r_*, and an imaginary part, *ԑ_i_*, where the real part represents the capacity of the material to reduce the speed of light and the imaginary part represents its capacity to efficiently absorb energy owing to polarization.

(7) $$\varepsilon =\varepsilon_{r}+i\varepsilon_{i}$$

*ԑ_r_* is determined from the refractive index, *n*, of the media ($\varepsilon_{r}=n^{2}-k^{2}$), and *ԑ_i_* is obtained from the extinction coefficient, k ($\varepsilon_{i}=2nk$) [10,97].

**Figure 16:**
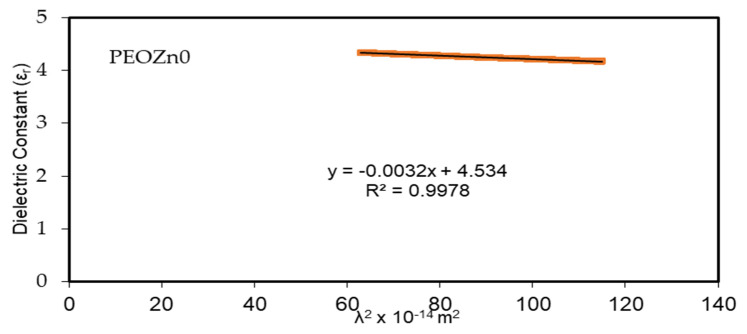

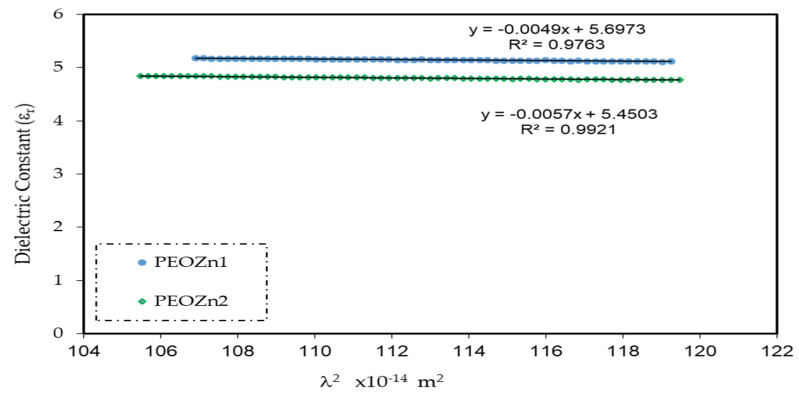
Optical dielectric constant vs. *λ*^2^ for PEO composites.

From the point of view of the Spitzer–Fan model, the dielectric constant of the material at a low frequency (long wavelengths), *ԑ_∞_*, is obtainable from correlations between the wavelength and refractive index [98]:

(8) $$\varepsilon_{r}=n^{2}-k^{2}=\varepsilon_{\infty }-\left(\frac{e^{2}}{4\;\pi^{2}C^{2}\varepsilon_{o}}\right)\times \left(\frac{N}{m^{*}}\right)\lambda^{2}$$

where the charge of an electron is *e*, the speed of light is denoted by *c*, *ԑ_o_* stands for the dielectric constant of free space, *N* is the localized density of a charge carrier, and m* stands for effective mass; all of the values are shown in Table 3 [99,100]. A straight-line result is obtained from plotting the values of *ԑ_r_* against *λ*^2^ in the visible spectrum range, as seen in Figure 16. The *ԑ*_∞_ and *N*/m* values, sequentially from the slope and intercept of the line of *ԑ_r_* versus λ^2^, are obtained using the constants listed in Table 3. An illustration of the values of *ԑ_∞_* and *N*/m* that are gained from Equation (8) is presented in Table 4.

The quantitative values in Table 4 imply that as doping concentration increases the localized density of state *N*/m* for the pure PEO sample increases from 3.89 × 10^55^ to 6.93 × 10^55^ atoms/m^3^, with the *ԑ_∞_*value increasing from 4.54 to 5.4503, showing that the growing of free charge carriers has strongly contributed to the process of polarization. The values expected for the localized density of states *N*/m* in Equation (8) of the current research are equivalent with those reported in the literature [54,101,102].

### 4.4. Tauc’s Approach for Band Gap Study

**Figure 17:**
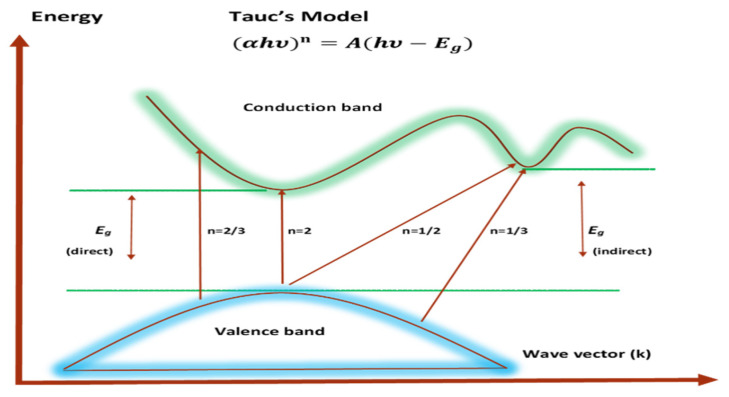
Types of electronic transitions [96].

**Figure 18:**
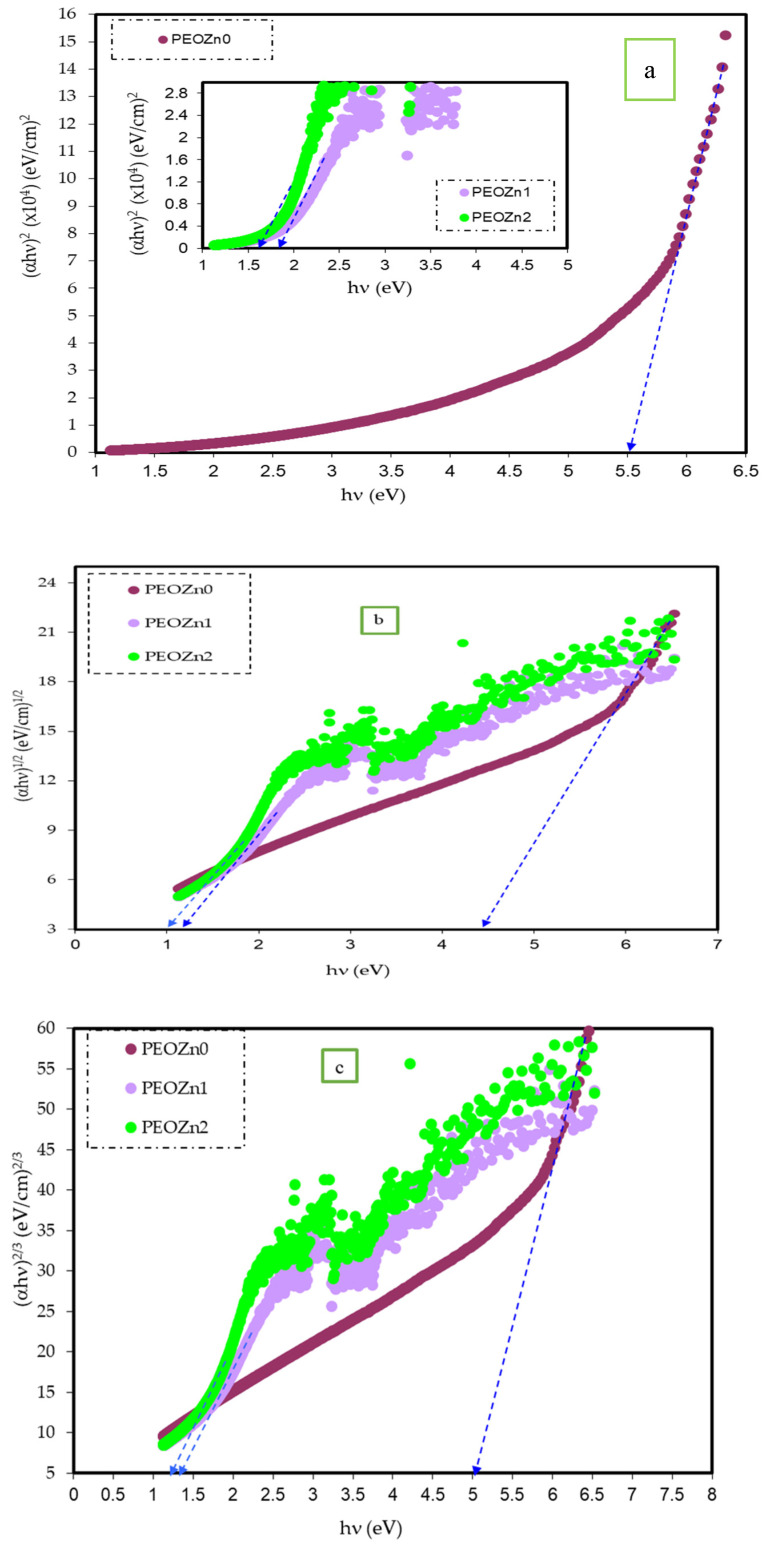

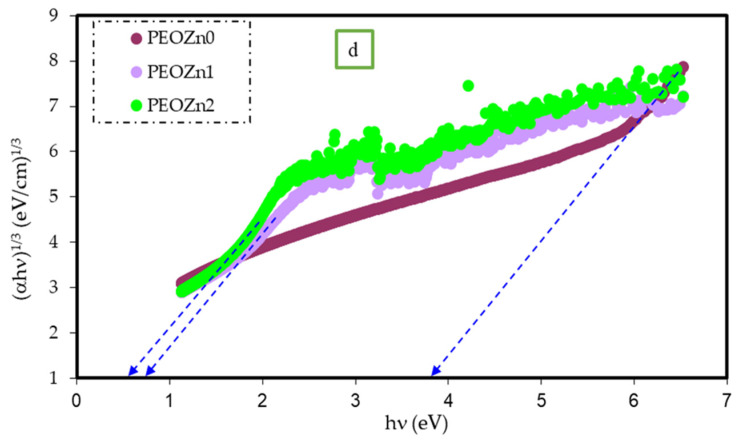
(**a**–**d**) The plot of ($\alpha hv{)}^{n}$ against the photon energy (*hυ*) of the PEO films for (**a**) *n* = 2, (**b**) *n* = 1/2, (**c**) *n* = 2/3, and (**d**) *n* = 1/3.

**Figure 19:**
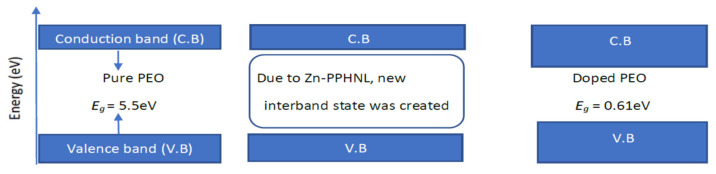
The effect of the metal complex on band gap reduction.

Broadband beams transmit through polymer films; band-to-band transitions occur by obeying particular selection rules and are characterized by a sharp increase in the fundamental absorption area [103,104]. According to the band structure of the materials, the transitions are divided into four different types [104]. Regarding amorphous semiconductors with indirect transitions, there is no conservation of electronic momentum when moving from the valence to the conduction band [105]. The following relationship provides the absorption coefficient for direct band gap materials [106]:

(9) $${\left(\alpha h\upsilon \right)}^{n}=B\left(h\upsilon -E_{g}\right)$$

where *hυ* stands for photon energy, *B* is a constant, and *E_g_* stands for the OBG energy, while the coefficient *n* defines the type of electronic transitions that lead to absorption [107]. Depending on the types of transition, the coefficient *n* can be 2, 2/3,1/2, or 1/3, corresponding to direct allowed, direct forbidden, indirect allowed, and indirect forbidden transitions, respectively, as shown clearly in Figure 17 [96]. The intercept of linear portions of the ($\alpha hv{)}^{n}$ of Figure 18a–d against the axis of photon energy, *hυ*, can be used to determine the value of *E_g_*. The determination of OBGs is crucial for comprehending the electrical behavior of a semiconductor and its practical interest [104]. The values of the OBGs of both transitions, allowed direct (*n* = 2) and forbidden direct (*n* = 2/3), are shown in Table 5. The materials of the amorphous phase and the band edges are influenced by the contribution of the various orbitals of both the metal complex and the ligand. As a consequence, the prediction as to whether the type of band is direct or indirect is a complicated task [108]. Thus, depending on the choices of *n*, many figures can be displayed by using the basic absorption equation (Equation (9)); however, only using Equation (9) makes it impossible to define the kind of electron transition. The complex dielectric function, ɛ*, needs to be examined in order to correctly classify the kind of electron transition. In order to calculate band structure in a relatively precise manner, the spectra of optical dielectric loss have to be carefully compared with Tauc’s plots. From such a comparison, the optical transition types for each solid film can be determined. In addition, the evolution of disorder in the polymer samples that results from the modification of a polymer’s structure can also be used to explain the OBG reduction [109,110]. The reduction in OBG results from the creation of new localized energy states in the bandgap between the VB and the CB [20]. Quantitatively, the declining of *E_g_* magnitudes as the Zn-PPHNL is increased is shown in Table 5 and Figure 19. By comparing the *E_g_* values in Tauc’s model (Figure 18a–d) with the energies from the optical dielectric loss as can be seen in later section, the types of transitions can be determined, such that for PEOZn0 it is direct allowed (*n* = 2), for PEOZn1 it is indirect allowed (*n* = 1/2), and for PEOZn2 it is an indirect forbidden (*n* =1/3) transition.

This study addresses creating polymer composites with small OBGs that close to semiconductor or conductive polymer materials. The reduction in the *E_g_* from 5.5 eV to 0.6 eV is achieved, verifying the hypothesis that green metal complexes can modify insulating polymers to semiconducting polymer composites. In general, polymer-based materials could show the property of declining OBGs by adding metal-based fillers. Table 6 shows the OBGs of different types of polymer composites with different fillers and dopants. Furthermore, the metal complex causes a more remarkable drop in the OBG compared to other types of fillers. The metal–PPHNL complexes contain several N-H and O-H, which in turn leads to interactions between the functional groups of metal–PPHNL complexes, with the polymer chains causing a drop in the gap between the valence and conduction bands of the doped polymer material. Based on the results of band gap reduction in the current work, Zn-PPHNL may introduce tremendous localized densities of states into the band gap region, and thus they overlap and reduce the band gap region in which the electrons should transfer from the VB to the CB. Moreover, it can be concluded that Zn-PPHNL is more influential than irradiation and ceramic nanoparticles, NPs, to manipulate the optical band gap of weakly polar polymers, such as PEO. Extra research is required to establish the fact that metal–PPHNL complexes are outstanding in reducing the band gap of weakly polar polymers.

### 4.5. Dielectric Function Study

Fundamental optical behaviors are explained through the use of the transverse dielectric function, the latter based on the momentum transfer, *q*, in the light–matter interaction and the transfer of energy. The total dielectric function is expressed as $\varepsilon^{*}=\varepsilon_{r}+i\varepsilon_{i}$, where *ε_r_* is the real part and depends on frequency [119] while the imaginary part, $\varepsilon_{i}$, describes how rapidly a medium absorbs electromagnetic waves [120]. Band gap investigations need to consider the quantum state of a material properly in order to be described more accurately, particularly the evaluation of the complex dielectric function, ɛ*. This parameter plays a crucial rule in the optical properties of matter and explains how a material’s electron density responds to the applied electromagnetic field [121].

In fact, the microscopic theory of the dielectric function applies a semiclassical method to establish the Hamiltonian function, describing the interaction between an incident electromagnetic field and Bloch electrons inside optical media. The modern description of electrons is via quantum mechanical (Bloch) wave functions; contrarily, the electromagnetic field is treated classically. This method is implemented since it is not as difficult as a total quantum mechanical approach, where an electromagnetic wave is considered as being quantized into photons. Nonetheless, it is more comprehensible and avoids sophisticated method of calculation [122]. The optical dielectric loss, *ε_i_*, is strongly associated with the electronic structure, in particular the localized density of states. Equation (10) is a fundamental relationship of the components of a matrix between the occupied and the unoccupied wave functions based on the selection rules [121]. In particular, the formula that essentially links the imaginary part, *ɛ_i_*, with the band structure is yielded from the electron–radiation interaction of the Hamiltonian function, *H_eR_*, which also describes how a charges move in the presence of an electromagnetic field inside a material [121]:

(10) $${ℋ}_{eR}=\frac{e}{mc}A\left(r,\;t\right)\cdot P$$

where *A*(*r*, *t*) is denoted as the vector potential and *P* is expressed as the momentum that conjugates to the vector of position. For semiconductor materials the momentum matrix elements of the electron enter directly into the *K·P* approach of band structure determination. There are various methods with which to find a semiconductor dielectric function from ${ℋ}_{eR}$. The most straightforward method is to assume that *A*(*r*, *t*) is small enough that time-dependent perturbation theory can be used (as represented in Fermi’s golden rule) to determine the transition probability (R) per unit volume for an electron in the valence band state |*V*⟩(with energy, *E_v_*, and wavevector, *k*_v_) to the conduction band *|C*⟩ (with equivalent energy, *E*c, and wavevector, *k*c). The electric dipole transition probability, R, for absorption photon per unit of time is expressed as follows:

(11) $$R=\frac{2\pi }{\hbar }{\left(\frac{e}{m\omega }\right)}^{2}{\left|\frac{E\left(\omega \right)}{2}\right|}^{2}\sum_{k}{\left|P_{CV}\right|}^{2}\;\delta (E_{c}(k_{c})-E_{V}(k_{v})-\;\hbar \;\omega )$$

In the above, the symbols for the incident photon frequency, Planck’s constant, effective mass, and electron charge are, respectively, *ω*, *ℏ*, *m*, and *e*.

Multiplying the transition probability per unit volume by the photon energy yields the power loss, *P*, by the field due to the medium absorption:

(12) P=Rℏω

The power loss can alternatively be stated in terms of the medium (absorption coefficient), *α*, or *ɛ_i_* by considering that the rate of the declining energy of incident beam per unit volume is given by *dI*/*dt*; *I* denotes the incident beam intensity:

(13) $$-\frac{dI}{dt}=-\left(\frac{dI}{Dx}\right)\left(\frac{dx}{dt}\right)=\frac{c}{n}\alpha I=\frac{\varepsilon_{i}\omega I}{n^{2}}$$

The density of energy, *I*, is linked to the amplitude of field as follows:

(14) $$I=\frac{n^{2}}{8\pi }{\left|E\left(\omega \right)\right|}^{2}$$

Then, we can obtain the following:

(15) $$\varepsilon_{i}\left(\omega \right)=\frac{1}{4\pi \varepsilon_{ο}}{\left(\frac{2\pi e}{m\omega }\right)}^{2}\underset{k}{\sum }{\left|P_{CV}\right|}^{2}\;\delta \left(E_{c\;}\left(k\right)-E_{V}\left(k\right)-\hbar \omega \right)$$

where *ω* expressed the incident photon, *εₒ* is the permittivity of the vacuum, and *e* is the electron charge. From a quantum mechanics point of view, Equation (15) indicates that the imaginary part’s optical dielectric function, *ɛ_i_*, is related to the band structure $\delta (E_{c\;}\left(k\right)-E_{V}\left(k\right)-\hbar \omega$, the delta function obtained in Fermi’s golden rule. This outcome articulates the fact that the electron absorbs the photon energy and is excited from the valence band into the conduction band [121]. The fundamental theory of an optical dielectric constant originates from a complex frequency function; it requires large-scale computation to determine dielectric constants [96,123]. The following equation illustrates how utilizing the extinction coefficient and refractive index in the calculation makes it simple to experimentally estimate the optical dielectric loss, *ε_i_* [13]:

(16) $$\varepsilon_{i}=2nk$$

It is challenging to determine whether the band will be direct or indirect when using Tauc’s model [108]. According to earlier theoretical investigations, there is a close connection between the optical dielectric function, $\varepsilon^{*}=\varepsilon_{r}+i\varepsilon_{i}$, and the band structure of semiconductor and insulating materials [124,125]. After the extrapolation of the linear component of the plot of *ε_i_* against photon energy, *hv*, the intercept is a useful way for calculating the OBG [111]. In reality, ε* shows the properties of the medium and how it responds to electromagnetic waves passing through it. The dielectric loss characterizes actual transitions between the occupied and unoccupied wave functions (electronic states) [124,126].

**Figure 20:**
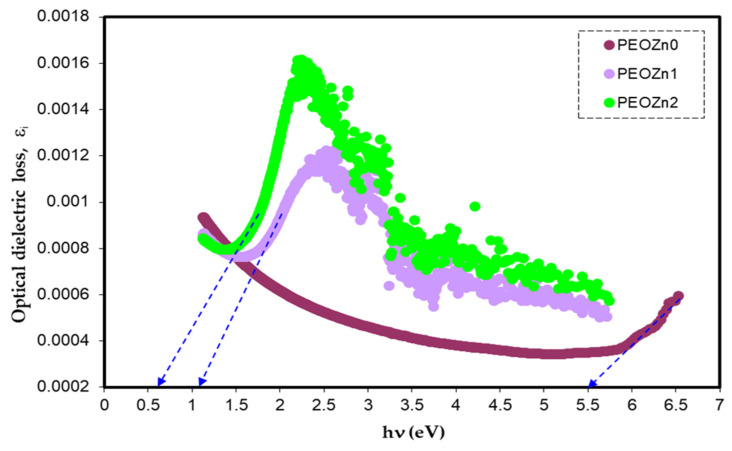
Optical dielectric loss vs. photon energy.

Simple equations can be used to calculate ɛ*, which is connected to the refractive index and extinction coefficient. Figure 20 illustrates the optical dielectric loss against photon energy for PEO samples. 

Previous research established that the appearance of peaks in the dielectric loss, *ε_i_*, are closely linked to interband transitions [127,128]. It has been noted that in amorphous or semicrystalline substances the band edges encompass associations from the various orbitals of the metal complex and the ligand, making it harder to identify if the band will be direct or indirect [120]. In order to accurately identify the sort of electronic transition, this work uses optical dielectric loss. Moreover, it has been proven that optical dielectric loss accurately depicts how an incident photon reacts to electronic transition [17,113]; therefore, the excitation energy of an electron from the valence to the conduction bands is determined from the extrapolation of the linear part of optical dielectric loss on the axis of photon energy. Additionally, the band gap is actually represented from this energy. In Tauc’s semiempirical approach, the transition process is represented by *n*, which can have one of the following values: 1/2, 3, 3/2, or 2, dependent on how the transitions of electrons are made from the valence band to the conduction band [108]. The kinds of electronic transition be assumed if the band gap calculated using Tauc’s model agrees with the one calculated by optical dielectric loss. This method for ascertaining materials’ band gap and kind of electronic transition is time-consuming, but it includes significant physics information. The electronic transition types can be identified from comparisons of the plots using Tauc’s equation (Figure 18, Figure 19 and Figure 20) of optical dielectric loss. From the comparisons it is convenient to argue that the kind of electronic transition in PEOZn0 is direct allowed (*n* = 2), for PEOZn1 it is indirect allowed *(n* = 1/2), and for PEOZn2 it is an indirect forbidden *(n* = 1/3) transition, which implies that the optical dielectric function is an efficient technique for examining the band structures of materials. The design of a polymer composite with a low cost and improved optical properties is a topic of great interest. In this domain, wide-band-gap PEO is one of the noteworthy and commonly used thermoplastic polymers; in comparison with conductive polymers it is cheap and stable. In neat PEO the crystalline state is what explains why the direct transitions are most likely to occur. In crystalline materials the top of the VB coincides with the bottom of the CB, while in amorphous materials this would not happen. In neat PEO the crystalline domains which are higher than amorphous phases may be responsible for the dominancy of direct transition. In the XRD section it was found that the amorphous phase increased in PEO: Zn-PPHNL composites compared to pure PEO. The optical micrograph (see Figure 10) clearly showed that neat PEO exhibits spherulites with big sizes, ascribed to the crystalline structure, while these spherulites were destroyed to small sizes and dark regions attributed to amorphous domains were dominant. In amorphous materials it is difficult to observe direct transition due to the disorder distribution of the valence and conduction bands. Su and Zhou observed the impact of crystallization on the enhancement of the optical and mechanical properties of PCCE polymer [129]. From the above discussion it can be emphasized that materials’ structures will greatly affect the optical and electrical properties.

## 5. Conclusions

A green chemistry strategy is followed in the preparation of PEO films with various amounts of the Zn-PPHNL metal complex. A black tea extract solution was used to synthesize Zn-PPHNL. This approach has recently drawn a great deal of attention in the research field associated with polymer composites, as it is cost-effective and environmentally friendly. Based on the quantitative results, an additive green metal complex remarkably reduces the optical band gap of polar polymers, which could be considered asignificant step forward to more diverse optoelectronics applications. An FTIR analysis revealed that BTLs have a lot of functional groups and conjugated double bonds, including O-H, C-H, C=O, C=C, C-O, C-N, and N-H, which could change the properties of PEO polymers significantly. Moreover, the FTIR results confirmthat while the formation of a PEO–metal complex’s, as well as a Zn-PPHNL complex’s, concentration in the samples increased, the position and intensity of the peaks somewhat varied. Additionally, several characteristic peaks of the Zn-PPHNL complex almost completely vanished due to Zn^2+^ ions’ attachment to the PPHNL, which results in a reduction in their vibration, increasing their weight. The XRD of the Zn-PPHNL and PEO composite revealed the enhancing of the amorphous phase of polymer films, such that by increasing Zn-PPHNL relative to PEO the relative XRD intensity of pure PEO declines. The OM micrographs of PEO films indicate that crystallinity decreases as the concentration of Zn-PPHNL increases, ascribed to the splitting of the spherulite structure into small entities and displaying dark spots on the surface of polymer films, which suggest that the amorphous phase was promoted.

The absorption of Zn-PPHNL enhances at the UV region because of ligand binding between tea PPHNL and Zn cations. UV spectroscopy was used to evaluate optical parameters, such as the refractive index (*n*), absorption edge, dielectric loss (*ε_i_*), dielectric constant (*ε_r_*), and bandgap energy (*E_g_*). Accordingly, the absorption edge changed from 5.25 eV for PEOZn0 to 1.1 eV for PEOZn samples. Additionally, the refractive index significantly increases from 2.05 to about 2.27 as a result of adding Zn-PPHNL to PEO. Moreover, it can be seen that as the Zn-PPHNL concentration in PEO films increased, the charge carriers (*N*/m*) values change from 3.89 × 10^55^ to 6.93 × 10^55^ atoms/m^3,^ and the value of *ԑ*_∞_ increases from 4.54 to 5.4503, showing that the increase in free charge carriers has significantly engaged in the polarization process. The W–D single-oscillator model was used to compute the oscillator dispersion energy, *E_d_*, and average oscillator energy, *E_o_*. Both *E_d_* and *E_o_* values have been strengthened from 20.48618 and 6.508456 to 10.04058 and 3.083462, respectively, by increasing the amount of the Zn-PPHNL complex solution. The OBG value decreases from 5.5 eV to 0.6 eV as the Zn-PPHNL insertion is increased. By comparing the values of *E_g_* corresponding to Tauc’s model with the energy from the dielectric loss, the types of electron transitions can be figured out: for PEOZn0 it is direct allowed (*n* = 2), for PEOZn1 it is indirect allowed (*n*= 1/2), while for PEOZn2 it is an indirect forbidden (*n* = 1/3) transition. These results support the argument that the crystalline dominancy in pure PEO is responsible for the direct allowed transition of electrons from the VB to the CB, while amorphous enrichment in the doped films is answerable for the prevailing indirect and forbidden transitions.

## Figures and Tables

**Table 1:** Absorption edge values for PEO films.

Sample Code	Absorption Edge (eV)
PEOZn0	5.25
PEOZn1	1.35
PEOZn2	1.1

**Table 2:** Empirical W–D single-oscillator model for calculating optical bandgap energy.

Films	*E_d_*	*E_o_*	*n_o_*
PEOZn0	20.48618	6.508456	4.147624
PEOZn1	12.56535	3.430341	4.663004
PEOZn2	10.04058	3.083462	4.256268

**Table 3:** Various physical parameters used for the calculation of *N*/m* for the prepared PEOZn-PPHNL.

Physical Parameter	Value
Mass of electron (*m_e_*)	9.109 × 10^−31^ Kg
Charge of electron (*e*)	1.602 × 10^−19^ coulombs
Permittivity of free space (*ԑ_o_*)	8.85 × 10^−12^ F/m
*π*	3.14
Speed of light (*c*)	2.99 × 10^8^ m/s
Effective mass (m*)	10.566 × 10^−31^ Kg

**Table 4:** Optical dielectric parameters values expected for PEO composite films.

Film	*N*/m* × 10^55^ (m^3^/kg)	*ɛ_∞_*
PEOZn0	3.89	4.54
PEOZn1	5.96	5.6973
PEOZn2	6.93	5.4503

**Table 5:** The *E_g_* from Tauc’s model versus photon energy and the optical dielectric loss, *ε_i_*, graph.

Sample Code	*E_g_* for (*αhv*)^2^	*E_g_* for (*αhv*)^2/3^	*E_g_* for (*αhv*)^1/2^	*E_g_* for (*αhv*)^1/3^	*E_g_* from *ɛ_i_*
PEOZn0	**5.5**	**5**	**4.42**	**3.8**	**5.5**
PEOZn1	**1.9**	**1.28**	**1.13**	**0.71**	**1.13**
PEOZn2	**1.67**	**1.23**	**1**	**0.6**	**0.61**

**Table 6:** Band gap energy, *E_g_*, values of various polymer composite systems.

Polymer Composite	Band Gap Energy *E_g_* (eV)	Reference
CS: silver nanoparticles	2.8–1.4	[111]
PS: (CaTiO_3_)	4.42–4.26	[112]
(CS: POZ): Zn^2+^-PPL complex	4.8–1.6	[113]
PVA: CeO_2_	6.34–6.09	[114]
PS:tin titanate nanoparticles	4.42–3.25	[115]
PVA: Al^3+^-metal complex	6.39–1.68	[54]
PVA: Co^2+^-polyphenol complex	5.8–1.82	[53]
PEO: CaTiO_3_nanoparticles	4.90–4.19	[116]
PVA: NaNO_3_	5.71–5.05	[117]
PVA: PbO_2_	6.32–4.33	[118]
PEO: Zn metal complex	5.5–0.61	Present work

## Data Availability

All the necessary data are presented.

## References

[1] Karman S.B., Diah S.Z.M., Gebeshuber I.C. (2014). Raw Materials Synthesis from Heavy Metal Industry Effluents with Bioremediation and Phytomining: A Biomimetic Resource Management Approach. Adv. Mater. Sci. Eng..

[2] Fu F., Wang Q. (2011). Removal of heavy metal ions from wastewaters: A review. J. Environ. Manag..

[3] Mohamed E.L.A., Hicham E.L.H. (2014). Synthesis and Characterization of caffeine Complexes [M (caf) 4X2] M = Ni (II), Cu (II), Zn (II), Cd (II) X = SCN-, CN-; caf: Caffeine. Res. J. Chem. Sci..

[4] Khalid S., Shahid M., Niazi N.K., Murtaza B., Bibi I., Dumat C. (2017). A comparison of technologies for remediation of heavy metal contaminated soils. J. Geochem. Explor..

[5] Zhai X., Li Z., Huang B., Luo N., Huang M., Zhang Q., Zeng G. (2018). Remediation of multiple heavy metal-contaminated soil through the combination of soil washing and in situ immobilization. Sci. Total Environ..

[6] Drynan J.W., Clifford M.N., Obuchowicz J., Kuhnert N. (2010). The chemistry of low molecular weight black tea polyphenols. Nat. Prod. Rep..

[7] van der Hooft J.J., Akermi M., Unlu F.Y., Mihaleva V., Roldan V.G., Bino R.J., de Vos R.C.H., Vervoort J. (2012). Structural annotation and elucidation of conjugated phenolic compounds in black, green, and white tea extracts. J. Agric. Food Chem..

[8] Lee L.S., Kim S.H., Kim Y.B., Kim Y.C. (2014). Quantitative analysis of major constituents in green tea with different plucking periods and their antioxidant activity. Molecules.

[9] Reto M., Figueira M.E., Filipe H.M., Almeida C.M.M. (2007). Chemical composition of green tea (*Camellia sinensis*) infusions commercialized in Portugal. Plant Foods Hum. Nutr..

[10] Aziz S.B. (2016). Modifying Poly(Vinyl Alcohol) (PVA) from Insulator to Small-Bandgap Polymer: A Novel Approach for Organic Solar Cells and Optoelectronic Devices. J. Electron. Mater..

[11] Aziz S.B., Abdullah O.G., Hussein A.M., Ahmed H.M. (2017). From insulating PMMA polymer to conjugated double bond behavior: Green chemistry as a novel approach to fabricate small band gap polymers. Polymers.

[12] Kotrba P., Mackova M., Macek T. (2011). Microbial Biosorption of Metals.

[13] MBrza A., Aziz S.B., Anuar H., Al Hazza M.H.F. (2019). From green remediation to polymer hybrid fabrication with improved optical band gaps. Int. J. Mol. Sci..

[14] Bauer R.E., Grimsdale A.C., Müllen K. (2005). Optical Properties of Hybrid Organic-Inorganic Materials and their Applications. Anion. Sens..

[15] Huang J., Yin Z., Zheng Q. (2011). Applications of ZnO in organic and hybrid solar cells. Energy Environ. Sci..

[16] Aziz S.B., Abdulwahid R.T., Rsaul H.A., Ahmed H.M. (2016). In situ synthesis of CuS nanoparticle with a distinguishable SPR peak in NIR region. J. Mater. Sci. Mater. Electron..

[17] Saeed C.O., Qader A.A., Aziz S.B. (2022). Low cost novel PEO based nano-composite for semiconductor and He–Ne lasers beam attenuation: Structural and optical properties. Opt. Mater..

[18] Yakuphanoglu F., Barim G., Erol I. (2007). The effect of FeCl3 on the optical constants and optical band gap of MBZMA-co-MMA polymer thin films. Phys. B Condens. Matter..

[19] Abdullah O.G., Aziz S.B., Omer K.M., Salih Y.M. (2015). Reducing the optical band gap of polyvinyl alcohol (PVA) based nanocomposite. J. Mater. Sci. Mater. Electron..

[20] Aziz S.B., Hussein S., Hussein A.M., Saeed S.R. (2013). Optical Characteristics of Polystyrene Based Solid Polymer Composites: Effect of Metallic Copper Powder. Int. J. Met..

[21] Kumar R., Ali S.A., Mahur A.K., Virk H.S., Singh F., Khan S.A., Avasthi D., Prasad R. (2008). Study of optical band gap and carbonaceous clusters in swift heavy ion irradiated polymers with UV-Vis spectroscopy. Nucl. Instrum. Methods Phys. Res. Sect. B Beam Interact. Mater. At..

[22] Han C.C., Shi W., Jin J. (2013). Morphology and Crystallization of Crystalline/Amorphous Polymer Blends. Encycl. Polym. Compos..

[23] Koduru H.K., Iliev M.T., Kondamareddy K.K., Karashanova D., Vlakhov T., Zhao X.Z., Scaramuzza N. (2016). Investigations on Poly (ethylene oxide) (PEO)-Blend based solid polymer electrolytes for sodium ion batteries. Journal of Physics: Conference Series.

[24] Cao Y.C., Xu C., Wu X., Wang X., Xing L., Scott K. (2011). A poly (ethylene oxide)/graphene oxide electrolyte membrane for low temperature polymer fuel cells. J. Power Source.

[25] Aziz S.B., Abdullah R.M. (2018). Crystalline and amorphous phase identification from the tanδ relaxation peaks and impedance plots in polymer blend electrolytes based on [CS:AgNt]x:PEO(x-1) (10 ≤ x ≤ 50). Electrochim. Acta.

[26] Huang C.I., Chen J.R. (2001). Crystallization and chain conformation of semicrystalline and amorphous polymer blends studied by wide-angle and small-angle scattering. J. Polym. Sci. Part B Polym. Phys..

[27] Yang R., Zhang S., Zhang L., Liu W. (2013). Electrical properties of composite polymer electrolytes based on PEO-SN-LiCF3SO3. Int. J. Electrochem. Sci..

[28] Kim M., Lee L., Jung Y., Kim S. (2013). Study on ion conductivity and crystallinity of composite polymer electrolytes based on poly(ethylene oxide)/poly(acrylonitrile) containing nano-sized Al2O3 Fillers. J. Nanosci. Nanotechnol..

[29] Rajeh A., Morsi M.A., Elashmawi I.S. (2019). Enhancement of spectroscopic, thermal, electrical and morphological properties of polyethylene oxide/carboxymethyl cellulose blends: Combined FT-IR/DFT. Vacuum.

[30] Abdelrazek E.M., Abdelghany A.M., Badr S.I., Morsi M.A. (2018). Structural, optical, morphological and thermal properties of PEO/PVP blend containing different concentrations of biosynthesized Au nanoparticles. J. Mater. Res. Technol..

[31] Wen S.J., Richardson T.J., Ghantous D.I., Striebel K.A., Ross P.N., Cairns E.J. (1996). FTIR characterization of PEO + LiN(CF3SO2)2 electrolytes. J. Electroanal. Chem..

[32] Bandara T.M.W.J., Karunathilaka D.G.N., Ratnasekera J.L., De Silva L.A., Herath A.C., Mellander B.E. (2017). Electrical and complex dielectric behaviour of composite polymer electrolyte based on PEO, alumina and tetrapropylammonium iodide. Ionics.

[33] Zhou H., Fu H., Wu X., Wu B., Dai C. (2020). Discrimination of tea varieties based on FTIR spectroscopy and an adaptive improved possibilistic c-means clustering. J. Food Process. Preserv..

[34] Verma D., Khan F. (2015). Corrosion Inhibition of High Carbon Steel in Phosphoric Acid Solution by Extract of Black Tea. Adv. Res..

[35] Loo Y.Y., Chieng B.W., Nishibuchi M., Radu S. (2012). Synthesis of silver nanoparticles by using tea leaf extract from *Camellia sinensis*. Int. J. Nanomed..

[36] Szymczycha-Madeja A., Welna M., Zyrnicki W. (2013). Multi-element analysis, bioavailability and fractionation of herbal tea products. J. Braz. Chem. Soc..

[37] Begum N.A., Mondal S., Basu S., Laskar R.A., Mandal D. (2009). Biogenic synthesis of Au and Ag nanoparticles using aqueous solutions of Black Tea leaf extracts. Colloids Surf. B Biointerfaces.

[38] Tao P., Li Y., Rungta A., Viswanath A., Gao J., Benicewicz B.C., Siegela R.W., Schadler L.S. (2011). TiO2 nanocomposites with high refractive index and transparency. J. Mater. Chem..

[39] Chaudhuri B., Uddin M.J., Chaudhuri B., Pramanik K., Middya T.R. (2012). Black tea leaf extract derived Ag nanoparticle-PVA composite film: Structural and dielectric properties. Mater. Sci. Eng. B Solid-State Mater. Adv. Technol..

[40] Senthilkumar S.R., Sivakumar T. (2014). Green tea (*Camellia sinensis*) mediated synthesis of zinc oxide (ZnO) nanoparticles and studies on their antimicrobial activities. Int. J. Pharm. Pharm. Sci..

[41] Hao R., Li D., Zhang J., Jiao T. (2021). Green synthesis of iron nanoparticles using green tea and its removal of hexavalent chromium. Nanomaterials.

[42] Dubey S.P., Sillanpaa M., Varma R.S. (2017). Reduction of hexavalent chromium using Sorbaria sorbifolia aqueous leaf extract. Appl. Sci..

[43] Ó’Coinceanainn M., Astill C., Schumm S. (2003). Potentiometric, FTIR and NMR studies of the complexation of metals with theaflavin. Dalt. Trans..

[44] Huang L., Weng X., Chen Z., Megharaj M., Naidu R. (2014). Synthesis of iron-based nanoparticles using oolong tea extract for the degradation of malachite green. Spectrochim. Acta-Part A Mol. Biomol. Spectrosc..

[45] Li X., Zhang Y., He Y. (2016). Rapid detection of talcum powder in tea using FT-IR spectroscopy coupled with chemometrics. Sci. Rep..

[46] Ucun F., Sa A., Güçlü V. (2007). Molecular structures and vibrational frequencies of xanthine and its methyl derivatives (caffeine and theobromine) by ab initio Hartree-Fock and density functional theory calculations. Spectrochim. Acta-Part A Mol. Biomol. Spectrosc..

[47] BGoodman A., Severino J.F., Pirker K.F. (2012). Reactions of green and black teas with Cu(ii). Food Funct..

[48] Zielinski A.A.F., Haminiuk C.W.I., Alberti A., Nogueira A., Demiate I.M., Granato D. (2014). A comparative study of the phenolic compounds and the in vitro antioxidant activity of different Brazilian teas using multivariate statistical techniques. Food Res. Int..

[49] Kolayli S., Ocak M., Küçük M., Abbaso R. (2004). Does caffeine bind to metal ions?. Food Chem..

[50] Brza M.A., Aziz S.B., Anuar H., Ali F., Dannoun E.M., Saeed S.R., Mohammed S.J., Abdulwahid R.T. (2021). Green coordination chemistry as a novel approach to fabricate polymer:Cd(II)-complex composites: Structural and optical properties. Opt. Mater..

[51] Brza M.A., Aziz S.B., Anuar H., Ali F., Dannoun E., Mohammed S.J., Abdulwahid R.T., Al S. (2020). Tea from the drinking to the synthesis of metal complexes and fabrication of PVA based polymer composites with controlled optical band gap. Sci. Rep..

[52] Aziz S.B., Nofal M.M., Brza M.A., Sadiq N.M., Dannoun E.M.A., Ahmed K.K., Al-Saeedi S.I., Hussen S.A., Hussein A.M. (2022). Innovative Green Chemistry Approach to Synthesis of Sn2+-Metal Complex and Design of Polymer Composites with Small Optical Band Gaps. Molecules.

[53] Nofal M.M., Aziz S.B., Hadi J.M., Karim W.O., Dannoun E.M., Hussein A.M., Hussen S.A. (2021). Polymer composites with 0.98 transparencies and small optical energy band gap using a promising green methodology: Structural and optical properties. Polymers.

[54] Aziz S.B., Nofal M.M., Ghareeb H.O., Dannoun E.M.A., Hussen S.A., Hadi J.M., Ahmed K.K., Hussein A.M. (2021). Characteristics of poly(Vinyl alcohol) (PVA) based composites integrated with green synthesized Al3+-metal complex: Structural, optical, and localized density of state analysis. Polymers.

[55] Yang Z., Peng H., Wang W., Liu T. (2010). Crystallization behavior of poly(ε-caprolactone)/layered double hydroxide nanocomposites. J. Appl. Polym. Sci..

[56] Ramesh S., Yuen T.F., Shen C.J. (2008). Conductivity and FTIR studies on PEO-LiX [X: CF3SO3-, SO42-] polymer electrolytes. Spectrochim. Acta-Part A Mol. Biomol. Spectrosc..

[57] Chu P.P., Reddy M.J., Tsai J. (2004). Structural and transport characteristics of polyethylene oxide/phenolic resin blend solid polymer electrolytes. J. Polym. Sci. Part B Polym. Phys..

[58] Patil S.U., Yawale S.S., Yawale S.P. (2014). Conductivity study of PEO-LiClO4 polymer electrolyte doped with ZnO nanocomposite ceramic filler. Bull. Mater. Sci..

[59] Senak L., Davies M.A., Mendelsohn R. (1991). A quantitative IR study of hydrocarbon chain conformation in alkanes and phospholipids: CH2 wagging modes in disordered bilayer and HII phases. J. Phys. Chem..

[60] Rao B.N.N., Suvarna R.P. (2016). A study on optical properties of poly (ethylene oxide) based polymer electrolyte with different alkali metal iodides. AIP Conference Proceedings.

[61] Sim L.H., Gan S.N., Chan C.H., Yahya R. (2010). ATR-FTIR studies on ion interaction of lithium perchlorate in polyacrylate/poly(ethylene oxide) blends. Spectrochim. Acta-Part A Mol. Biomol. Spectrosc..

[62] Soman V.V., Kelkar D.S. (2009). FTIR studies of doped PMMA-PVC blend system. Macromol. Symp..

[63] APalacios-Morillo A., Alcázar Á., De Pablos F., Jurado J.M. (2013). Differentiation of tea varieties using UV-Vis spectra and pattern recognition techniques. Spectrochim. Acta-Part A Mol. Biomol. Spectrosc..

[64] Srivastavaab A., Singha V., Aggarwat P., Schneeweiss F., Scherer U.W., Friedrichc W. (2010). Optical studies of insulating polymers for radiation dose monitoring. Indian J. Pure Appl. Phys..

[65] Marzuki A., Suryanti V., Virgynia A. (2017). Spectroscopic Study of Green Tea (*Camellia sinensis*) Leaves Extraction. IOP Conference Series: Materials Science and Engineering.

[66] Kumar K.R.P., Murali M.G., Udayakumar D. (2014). Synthesis and study of optical properties of linear and hyperbranched conjugated polymers containing thiophene and triphenylamine units. Des. Monomers Polym..

[67] Koyuncu F.B., Sefer E., Koyuncu S., Ozdemir E. (2011). A new low band gap electrochromic polymer containing 2,5-bis-dithienyl-1H-pyrrole and 2,1,3-benzoselenadiazole moiety with high contrast ratio. Polymer.

[68] López-Gutiérrez N., Romero-González R., Plaza-Bolaños P., Vidal J.L.M., Frenich A.G. (2015). Identification and quantification of phytochemicals in nutraceutical products from green tea by UHPLC-Orbitrap-MS. Food Chem..

[69] Pasrija D., Anandharamakrishnan C. (2015). Techniques for Extraction of Green Tea Polyphenols: A Review. Food Bioprocess Technol..

[70] Wang X., Huang J., Fan W., Lu H. (2015). Identification of green tea varieties and fast quantification of total polyphenols by near-infrared spectroscopy and ultraviolet-visible spectroscopy with chemometric algorithms. Anal. Methods.

[71] Hoag G.E., Collins J.B., Holcomb J.L., Hoag J.R., Nadagouda M.N., Varma R.S. (2009). Degradation of bromothymol blue by ‘greener’ nano-scale zero-valent iron synthesized using tea polyphenols. J. Mater. Chem..

[72] Aziz S.B., Marif R.B., Brza M.A., Hassan A.N., Ahmad H.A., Faidhalla Y.A., Kadir M.F.Z. (2019). Structural, thermal, morphological and optical properties of PEO filled with biosynthesized Ag nanoparticles: New insights to band gap study. Results Phys..

[73] Aziz S.B., Faraj M.G., Abdullah O.G. (2018). Impedance Spectroscopy as a Novel Approach to Probe the Phase Transition and Microstructures Existing in CS:PEO Based Blend Electrolytes. Sci. Rep..

[74] Saeed K., Ishaq M., Ilyas M. (2011). Preparation, morphology, and thermomechanical properties of coal ash/polyethylene oxide composites. Turk. J. Chem..

[75] Abdullah M., Lenggoro W., Okuyama K. (2004). Polymer Electrolyte Nanocomposites.

[76] Surov O.V., Voronova M.I., Afineevskii A.V., Zakharov A.G. (2018). Polyethylene oxide films reinforced by cellulose nanocrystals: Microstructure-properties relationship. Carbohydr. Polym..

[77] Aziz S.B., Hassan A.Q., Mohammed S.J., Karim W.O., FZKadir M., ATajuddin H., NMY Chan N. (2019). Structural and optical characteristics of pva:C-dot composites: Tuning the absorption of ultra violet (uv) region. Nanomaterials.

[78] Yin X., Liu J., Jäkle F. (2021). Electron-Deficient Conjugated Materials via p–π* Conjugation with Boron: Extending Monomers to Oligomers, Macrocycles, and Polymers. Chem.-A Eur. J..

[79] Hasegawa T., Ashizawa M., Hiyoshi J., Kawauchi S., Mei J., Bao Z., Matsumoto H. (2016). An ultra-narrow bandgap derived from thienoisoindigo polymers: Structural influence on reducing the bandgap and self-organization. Polym. Chem..

[80] Hareesh K., Sanjeev G., Pandey A.K., Rao V. (2013). Characterization of UV-irradiated Lexan polycarbonate films. Iran. Polym. J..

[81] Singh V., Mohan S., Singh G., Pandey P.C., Prakash R. (2008). Synthesis and characterization of polyaniline-carboxylated PVC composites: Application in development of ammonia sensor. Sens. Actuators B Chem..

[82] Elimat Z.M., Zihlif A.M., Avella M. (2008). Thermal and optical properties of poly(methyl methacrylate)/calcium carbonate nanocomposite. J. Exp. Nanosci..

[83] Aziz S.B., Ahmed H.M., Hussein A.M., Fathulla A.B., Wsw R.M., Hussein R.T. (2015). Tuning the absorption of ultraviolet spectra and optical parameters of aluminum doped PVA based solid polymer composites. J. Mater. Sci. Mater. Electron..

[84] Abdullah R.M., Aziz S.B., Mamand S.M., Hassan A.Q., Hussein S.A., Kadir M.F.Z. (2019). Reducing the crystallite size of spherulites in PEO-based polymer nanocomposites mediated by carbon nanodots and Ag nanoparticles. Nanomaterials.

[85] Aziz S.B. (2017). Morphological and optical characteristics of chitosan(1−x):Cuox (4 ≤ x ≤ 12) based polymer nano-composites: Optical dielectric loss as an alternative method for tauc’s model. Nanomaterials.

[86] Tippins H.H. (1970). Charge-transfer spectra of transition-metal ions in corundum. Phys. Rev. B.

[87] Sharma H., Sharma S.N., Singh G., Shivaprasad S.M. (2007). Studies of optical and structural properties of CdSe/polymer nanocomposites: Evidence of charge transfer and photostability. Colloid Polym. Sci..

[88] Yakuphanoglu F., Sekerci M., Balaban A. (2005). The effect of film thickness on the optical absorption edge and optical constants of the Cr(III) organic thin films. Opt. Mater..

[89] Aziz S.B., Rasheed M.A., Ahmed H.M. (2017). Synthesis of polymer nanocomposites based on [methyl cellulose] (1−x):(CuS)x (0.02 M ≤ x ≤ 0.08 M) with desired optical band gaps. Polymers.

[90] Yetisen A.K., Montelongo Y., Butt H. (2016). Rewritable three-dimensional holographic data storage via optical forces. Appl. Phys. Lett..

[91] Muhammad F.F., Sulaiman K. (2011). Photovoltaic performance of organic solar cells based on DH6T/PCBM thin film active layers. Thin Solid Film..

[92] Taha T.A. (2019). Optical properties of PVC/Al2O3 nanocomposite films. Polym. Bull..

[93] Wemple S.H., DiDomenico M. (1971). Behavior of the electronic dielectric constant in covalent and ionic materials. Phys. Rev. B.

[94] Ammar A.H. (2002). Studies on some structural and optical properties of Zn x Cd 1-x Te thin films. Appl. Surf. Sci..

[95] Benchaabane A., Ben Hamed Z., Kouki F., Abderrahmane Sanhoury M., Zellama K., Zeinert A., Bouchriha H. (2014). Performances of effective medium model in interpreting optical properties of polyvinylcarbazole:ZnSe nanocomposites. J. Appl. Phys..

[96] Aziz S.B., Brza M.A., Nofal M.M., Abdulwahid R.T., Hussen S.A., Hussein A.M., Karim W.O. (2020). A comprehensive review on optical properties of polymer electrolytes and composites. Materials.

[97] Ebnalwaled A.A., Thabet A. (2016). Controlling the optical constants of PVC nanocomposite films for optoelectronic applications. Synth. Met..

[98] Ali F.M., Kershi R.M., Sayed M.A., AbouDeif Y.M. (2018). Evaluation of structural and optical properties of Ce3+ ions doped (PVA/PVP) composite films for new organic semiconductors. Phys. B Condens. Matter..

[99] Spitzer W.G., Fan H.Y. (1957). Determination of optical constants and carrier effective mass of semiconductors. Phys. Rev..

[100] Kiselev A.I., Akashev L.A., Kononenko V.I. (2004). Effective electron mass in melts of aluminum, cerium, and Al-3 at.% Ce binary system. Tech. Phys..

[101] Alsaad A.M., Al-Bataineh Q.M., Ahmad A.A., Albataineh Z., Telfah A. (2020). Optical band gap and refractive index dispersion parameters of boron-doped ZnO thin films: A novel derived mathematical model from the experimental transmission spectra. Optik.

[102] Benchaabane A., Hajlaoui M.E., Hnainia N., Al-Tabbakh A., Zeinert A., Bouchriha H. (2020). Optical properties enhancement of hybrid nanocomposites thin films based on P3HT matrix and ZnO@SiO_2_ core-shell nanoparticles. Opt. Mater..

[103] Yakuphanoglu F., Sekerci M., Ozturk O.F. (2004). The determination of the optical constants of cu(ii) compound having 1-chloro-2,3-o-cyclohexylidinepropane thin film. Opt. Commun..

[104] Ahmed N.M., Sauli Z., Hashim U., Al-Douri Y. (2009). Investigation of the absorption coefficient, refractive index, energy band gap, and film thickness for Al0.11Ga0.89N by optical transmission method. Int. J. Nanoelectron. Mater..

[105] Vergara M.E.S., Rebollo A.O., Alvarez J.R., Rivera M. (2008). Molecular materials derived from MPc (M = Fe, Pb, Co) and 1,8-dihydroxiantraquinone thin films: Formation, electrical and optical properties. J. Phys. Chem. Solids.

[106] El-Nahass M.M., Farid A.M., Atta A.A. (2010). Structural and optical properties of Tris(8-hydroxyquinoline) aluminum (III) (Alq3) thermal evaporated thin films. J. Alloys Compd..

[107] Edukondalu A., Rahman S., Ahmmad S.K., Gupta A., Kumar K.S. (2016). Optical properties of amorphous Li2O–WO3–B2O3 thin films deposited by electron beam evaporation. J. Taibah Univ. Sci..

[108] Rodríguez A., Vergara M.E.S., Montalvo V.G., Ortiz A., Alvarez J.R. (2010). Thin films of molecular materials synthesized from C_32_H_20_N_10_M (M = Co, Pb, Fe): Film formation, electrical and optical properties. Appl. Surf. Sci..

[109] Rozra J., Saini I., Sharma A., Chandak N., Aggarwal S., Dhiman R., Sharma P.K. (2012). Cu nanoparticles induced structural, optical and electrical modification in PVA. Mater. Chem. Phys..

[110] Abdelaziz M. (2011). Cerium (III) doping effects on optical and thermal properties of PVA films. Phys. B Condens. Matter..

[111] SAziz B., Mamand S.M., Saed S.R., Abdullah R.M., Hussein S.A. (2017). New Method for the Development of Plasmonic Metal-Semiconductor Interface Layer: Polymer Composites with Reduced Energy Band Gap. J. Nanomater..

[112] Ezat G.S., Hussen S.A., Aziz S.B. (2021). Structure and optical properties of nanocomposites based on polystyrene (PS) and calcium titanate (CaTiO3) perovskite nanoparticles. Optik.

[113] Ahmed K.K., Hussen S.A., Aziz S.B. (2022). Transferring the wide band gap chitosan: POZ-based polymer blends to small optical energy band gap polymer composites through the inclusion of green synthesized Zn2+-PPL metal complex. Arab. J. Chem..

[114] Aziz S.B., Dannoun E.M.A., Tahir D.A., Hussen S.A., Abdulwahid R.T., Nofal M.M.M., Abdullah R.M., Hussein A., Brevik I. (2021). Synthesis of pva/ceo2 based nanocomposites with tuned refractive index and reduced absorption edge: Structural and optical studies. Materials.

[115] Hussein A.M., Dannoun E.M.A., Aziz S.B., Brza M.A., Abdulwahid R.T., Hussen S.A., Rostam S., Mustafa D.M.T., Muhammad D.S. (2020). Steps toward the band gap identification in polystyrene based solid polymer nanocomposites integrated with tin titanate nanoparticles. Polymers.

[116] Aziz S.B., Nofal M.M., Brza M.A., Hussein S.A., Mahmoud K.H., El-Bahy Z.M., Dannoun E.M.A., Kareem W.O., Hussein A.M. (2021). Characteristics of peo incorporated with CaTiO3 nanoparticles: Structural and optical properties. Polymers.

[117] Muhammad F.F., Aziz S.B., Hussein S.A. (2015). Effect of the dopant salt on the optical parameters of PVA:NaNO3 solid polymer electrolyte. J. Mater. Sci. Mater. Electron..

[118] Abdulwahid R.T., Abdullah O.G., Aziz S.B., Hussein S.A., Muhammad F.F., Yahya M.Y. (2016). The study of structural and optical properties of PVA:PbO2 based solid polymer nanocomposites. J. Mater. Sci. Mater. Electron..

[119] Soni A., Dashora A., Gupta V., Arora C.M., Rérat M., Ahuja B.L., Pandey R. (2011). Electronic and optical modeling of solar cell compounds CuGaSe 2 and CuInSe 2. J. Electron. Mater..

[120] Mahmood Q., Haq B.U., Yaseen M., Ramay S.M., Ashiq M.G.B., Mahmood A. (2019). The first-principle study of mechanical, optical and thermoelectric properties of SnZrO 3 and SnHfO 3 for renewable energy applications. Solid State Commun..

[121] Kreher K. (1997). Fundamentals of Semiconductors–Physics and Materials Properties. Z. Phys. Chem..

[122] Bouzidi C., Horchani-Naifer K., Khadraoui Z., Elhouichet H., Ferid M. (2016). Synthesis, characterization and DFT calculations of electronic and optical properties of CaMoO4. Phys. B Condens. Matter..

[123] Ravindra N., Ganapathy P., Choi J. (2007). Energy gap-refractive index relations in semiconductors-An overview (in press version with book). Infrared Phys. Technol..

[124] Rasul S.M., Saber D.R., Aziz S.B. (2022). Role of Titanium replacement with Pd atom on band gap reduction in the anatase Titanium Dioxide: First-Principles calculation approach. Results Phys..

[125] Qadr R.A., Saber D.R., Aziz S.B. (2022). Theoretical Investigations of Electronic and Optical Properties of Vanadium Doped Wurtzite Zinc Oxide from First Principle Calculation Method. Iraqi J. Phys..

[126] Hossain F.M., Sheppard L., Nowotny J., Murch G.E. (2008). Optical properties of anatase and rutile titanium dioxide: Ab initio calculations for pure and anion-doped material. J. Phys. Chem. Solids.

[127] Feng J., Xiao B., Chen J.C., Zhou C.T., Du Y.P., Zhou R. (2009). Optical properties of new photovoltaic materials: AgCuO_2_ and Ag_2_Cu_2_O_3_. Solid State Commun..

[128] Logothetidis S. (2003). Optical and electronic properties of amorphous carbon materials. Diam. Relat. Mater..

[129] Su B., Zhou Y.-G. (2019). Improvement of transparencies and mechanical properties of poly (cyclohexylene dimethylene cyclohexanedicarboxylate) parts using a compounding nucleating agent to control crystallization. Materials.

